# Cooperation between PRMT1 and PRMT6 drives lung cancer health disparities among Black/African American men

**DOI:** 10.1016/j.isci.2024.108858

**Published:** 2024-01-11

**Authors:** Pei-Ying Wu, Michelle Van Scoyk, Stephanie S. McHale, Chu-Fang Chou, Gregory Riddick, Kamran Farouq, Bin Hu, Vita Kraskauskiene, Jennifer Koblinski, Charles Lyons, Arjun Rijal, Vignesh Vudatha, Dongyu Zhang, Jose G. Trevino, Rachit D. Shah, Patrick Nana-Sinkam, Yong Huang, Shwu-Fan Ma, Imre Noth, Chanita Hughes-Halbert, Victoria L. Seewaldt, Ching-Yi Chen, Robert A. Winn

**Affiliations:** 1 Massey Comprehensive Cancer Center, Virginia Commonwealth University, Richmond, VA, USA; 2 Department of Pathology and Massey Comprehensive Cancer Center, Virginia Commonwealth University, Richmond, VA, USA; 3 Division of Surgical Oncology and Massey Comprehensive Cancer Center, Virginia Commonwealth University, Richmond, VA, USA; 4 Division of Cardiothoracic Surgery, Virginia Commonwealth University, Richmond, VA, USA; 5 Division of Pulmonary Disease and Critical Care Medicine, Virginia Commonwealth University, Richmond, VA, USA; 6 Division of Pulmonary and Critical Care, University of Virginia, Charlottesville, VA, USA; 7 Norris Comprehensive Cancer Center, University of Southern California, Los Angeles, CA, USA; 8 City of Hope Comprehensive Cancer Center, Duarte, CA, USA

**Keywords:** Health disparity, Molecular biology, Cancer

## Abstract

Lung cancer is the third most common cancer with Black/AA men showing higher risk and poorer outcomes than NHW men. Lung cancer disparities are multifactorial, driven by tobacco exposure, inequities in care access, upstream health determinants, and molecular determinants including biological and genetic factors. Elevated expressions of protein arginine methyltransferases (PRMTs) correlating with poorer prognosis have been observed in many cancers. Most importantly, our study shows that PRMT6 displays higher expression in lung cancer tissues of Black/AA men compared to NHW men. In this study, we investigated the underlying mechanism of PRMT6 and its cooperation with PRMT1 to form a heteromer as a driver of lung cancer. Disrupting PRMT1/PRMT6 heteromer by a competitive peptide reduced proliferation in non-small cell lung cancer cell lines and patient-derived organoids, therefore, giving rise to a more strategic approach in the treatment of Black/AA men with lung cancer and to eliminate cancer health disparities.

## Highlights

• PRMT1 and PRMT6 form a heteromer
• Disrupting PRMT1/PRMT6 heteromer reduces growth of NSCLC cells and LC PDOs
• PRMT1/PRMT6 heteromer recruits and catalyzes the arginine methylation of ILF2
• Arginine methylation inhibits the ubiquitination-mediated degradation of ILF2

## Graphical abstract

**Figure:**
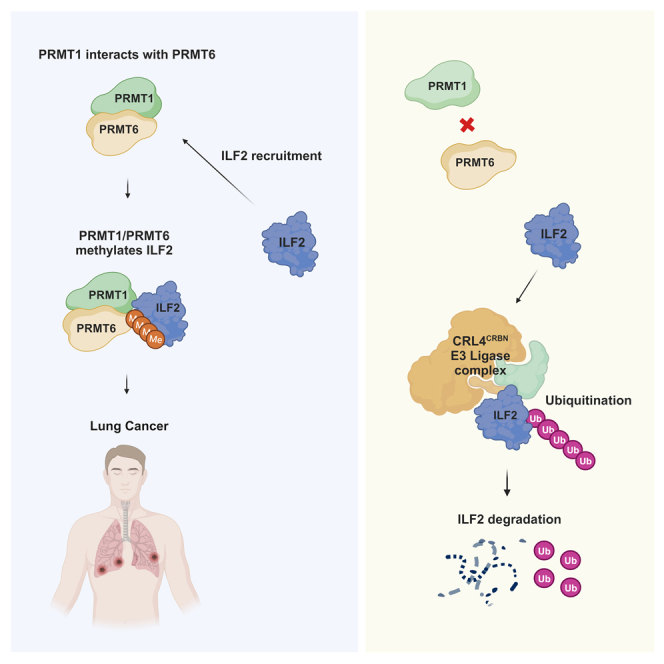

## Introduction

Lung cancer is the leading cause of cancer-related death in the world. Non-small cell lung cancer (NSCLC) accounting for approximately 85% of all lung cancer types includes lung adenocarcinoma (LUAD), lung squamous cell carcinoma (LUSC), and large cell carcinoma (LCC). Despite declines in incidence over the last decade, the five-year survival rate for NSCLC is approximately 26%.^1^ While survival has increased, Black/AA men in the United States have disproportionally higher incidence and mortality rates of lung cancer compared to NHW men.^2^^,^^3^ Lung cancer disparities in Black/AA men are multifactorial, driven by more than tobacco exposure, instead involving complex and poorly understood interactions between inequities in access to care, upstream determinants of health, and community stress.^2^^,^^4^ Additionally, biological factors are also believed to play critical roles in driving the disparities.^5^^,^^6^ However, several recent large-scale genomic studies have failed to identify significant differences in somatic mutations in lung cancer driver genes (e.g., *EGFR* and *KRAS*) and other genes important for cancer development in Black/AA versus NHW.^4^^,^^7^^,^^8^^,^^9^^,^^10^^,^^11^ These findings suggest that other complex genetic and/or epigenetic mechanisms may contribute to the differences in outcomes between Black/AA men and NHW men with lung cancer. Therefore, identifying biomarkers and/or critical genes to serve as early detection or novel therapeutics could reduce lung cancer incidence and mortality rates among Black/AA men.

Arginine methylation is a post-translational modification catalyzed by protein arginine methyltransferases (PRMTs). PRMTs are a family of enzymes known to methylate arginine residues on histones and non-histone proteins, resulting in type I asymmetrical dimethylarginine catalyzed by PRMT1, PRMT2, PRMT3, PRMT4, PRMT6, and PRMT8, type II symmetrical dimethylarginine catalyzed by PRMT5 and PRMT9, and type III monomethylarginine catalyzed by PRMT7.^12^ Subsequently, the methylation of downstream protein substrates affects transcription, splicing, translation, signal transduction, DNA damage and repair, and cancer development.^13^^,^^14^

PRMTs have gained considerable interest as many cancer types display elevated expression of PRMTs correlating with poorer prognosis.^12^^,^^15^ Supporting literature has shown that PRMTs play a role in many cancers (e.g., breast, hepatocellular, colorectal, gastric, bladder, leukemia, and neuroblastoma) including lung cancer.^16^^,^^17^ Because of their importance in cancer, several small molecule inhibitors that target the catalytic activity and/or substrate binding of PRMTs have been developed and entered clinical trials.^15^ The targeting of PRMTs has taken several forms that include the global inhibition of individual PRMTs by the targeting of the catalytic region, substrate competition, and dual inhibition of multiple PRMTs to increase synergistic effects.^15^^,^^18^^,^^19^^,^^20^ While these endeavors appear worthwhile, they all share significant challenges and limitations in cancer treatment due to a lack of selectivity and potency.^15^^,^^16^^,^^18^ Furthermore, the targeting of PRMT1 is potentially problematic since the full-body deletion of PRMT1 is embryonically lethal.^21^ Thus, developing alternative approaches, e.g., blocking co-factor recruitment, may better target a given PRMT isoform specifically without affecting other isoforms.

ILF2, also known as nuclear factor 45 (NF45), forms a heterodimer with ILF3 and interacts with other nuclear factors to regulate gene expression at both transcriptional and post-transcription levels.^22^^,^^23^^,^^24^ ILF2 is overexpressed in multiple myeloma to drive resistance to DNA damaging agents^24^ and facilitates nuclear export of mRNAs encoding pluripotency factors in human esophageal cancer.^25^ Mutations within the ILF2 promoter associated with increased expression were identified in a subset of lung adenocarcinoma without RTK/RAS/RAF pathway alterations.^26^ Although the dysregulation of ILF2 is involved in multiple cancer types, its role in lung cancer remains unclear.

Recent studies have shown that type I PRMTs play critical roles in lung cancer development. PRMT1, PRMT4, and PRMT6 are elevated in NSCLC tissue, and silencing the expression reduces cell proliferation.^27^^,^^28^ Among these three PRMTs, PRMT1 interacts with PRMT6^29^^,^^30^; however, the significance of the interactions is still unknown. Most importantly, whether any of these PRMTs drive lung cancer and health disparities in Black/AA men still remains unclear. In the present study, we aimed to test the hypothesis and determine the molecular mechanism of promoting lung cancer by PRMTs. We found higher PRMT6 expression in LUAD of Black/AA men versus NHW men. We demonstrated that PRMT1 and PRMT6 formed a heteromer, and breaking this heteromer with a peptide inhibitor reduced cell proliferation and viability in NSCLC cell lines and lung cancer organoids. We also uncovered ILF2 as a specific substrate for the PRMT1/PRMT6 heteromer, in which arginine methylation reduced the proteasome-mediated degradation of ILF2. Together, our results implicate that high PRMT6 expression cooperates with PRMT1 to drive higher incidence and mortality rates of lung cancer in Black/AA men. Targeting the heteromer could likely eliminate lung cancer health disparities.

## Results

### Higher PRMT6 expression is detected in lung adenocarcinoma of Black/AA men compared to NHW men

**Figure 1:**
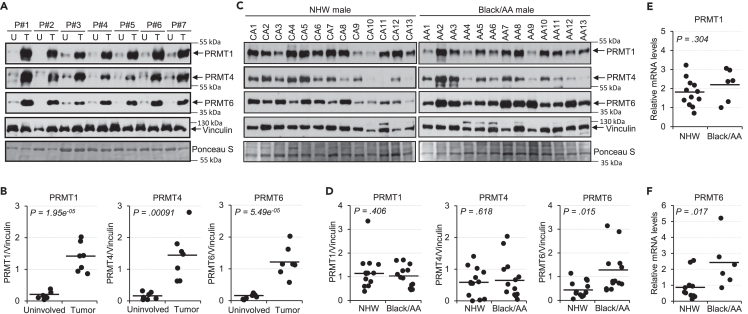
Expression of PRMT1, PRMT4, and PRMT6 is elevated in NSCLC tissue, and higher PRMT6 levels are detected in LUAD tissue of Black/AA men compared to NHW men (A) Extracts of uninvolved lung tissue and tumor tissue from the same patient were analyzed by immunoblotting using anti-PRMT1, anti-PRMT4, anti-PRMT6, or anti-vinculin. A portion of Ponceau S staining for equal protein loading is shown. (B) Quantification of signals in (A) is shown. Bars indicate the mean of all samples (two-tailed t-test for p value). (C) Extracts of LUAD tissue from NHW men and Black/AA men were analyzed by immunoblotting using anti-PRMT1, anti-PRMT4, anti-PRMT6, or anti-vinculin. A portion of Ponceau S staining for equal protein loading is shown. Vinculin blots here are the same ones in Figure S6D. (D) Quantification of signals in (C) is shown. Bars indicate the mean of all samples (two-tailed t-test for p value). (E and F) mRNA levels of PRMT1 (E) and PRMT6 (F) in LUAD tissue from NHW men and Black/AA men were analyzed by qRT-PCR. Bars indicate the mean of all samples (two-tailed t-test for p value). See also Figures S1 and S2.

To determine whether type I PRMTs play a role in lung cancer health disparities, we analyzed the expression levels in NSCLC using TCGA datasets and the association of expression with prognosis and outcomes. Consistent with previous studies,^27^^,^^28^ we found that PRMT1, PRMT4, and PRMT6 were up-regulated in LUAD and LUSC (Figures S1A–S1F) and high expression of these PRMTs was associated with shorter survival in LUAD (Figures S1G–S1I), but not in LUSC (Figures S1J–S1L) analyzed by Kaplan-Meier Plotter.^31^ There is no difference in PRMT1 and PRMT4 expression in LUAD between Black/AA and NHW in both men and women (Figures S2A–S2D). However, higher PRMT6 expression in LUAD of Black/AA men compared to NHW men (Figure S2E) was observed, but not in women (Figure S2F). No differential expression of PRMT6 was observed in LUSC of Black/AA men and women versus NHW men and women (Figures S2G and S2H). To further validate the observation, we analyzed the expression of PRMT1, PRMT4, and PRMT6 in fresh frozen lung tumor tissue and found elevated expression of all three PRMTs in tumor tissue compared to adjacent uninvolved tissue (Figures 1A and 1B). Most importantly, PRMT6 expression was higher in LUAD tissue of Black/AA men compared to NHW men (Figures 1C and 1D). PRMT6, but not PRMT1, mRNA levels were also higher in LUAD tissue of Black/AA men compared to NHW men (Figures 1E and 1F). Taken together, our results show that, among type I PRMTs, PRMT1, PRMT4, and PRMT6 are up-regulated and associated with worse outcomes in patients with LUAD. Higher PRMT6 levels in LUAD of Black/AA men may contribute to lung cancer health disparities observed in this particular cohort.

### PRMT1 and PRMT6 form a heteromer

**Figure 2:**
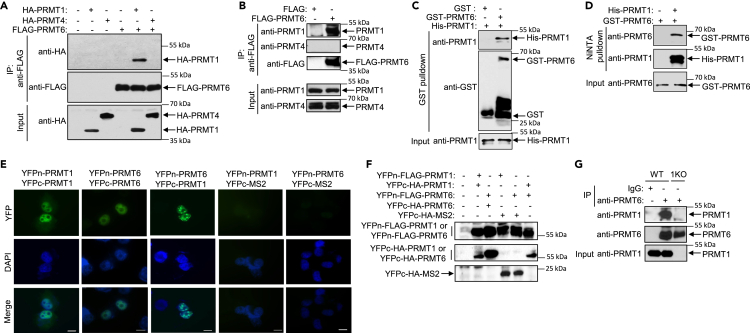
PRMT1 interacts with PRMT6 (A) H1299 cells were co-transfected with a control vector or a vector expressing FLAG-PRMT6 and vectors expressing HA-PRMT1 or HA-PRMT4. Cell extracts were subjected to anti-FLAG immunoprecipitation. The immunoprecipitates were analyzed by anti-HA or anti-FLAG immunoblotting. (B) H1299 cells were transfected with a control vector or a vector expressing FLAG-PRMT6. Cell extracts were subjected to anti-FLAG immunoprecipitation. The immunoprecipitates were analyzed by anti-PRMT1, anti-PRMT4, or anti-FLAG immunoblotting. (C) GST or GST-PRMT6 was incubated with His-PRMT1. The reactions were subjected to GST pulldown followed by anti-PRMT1 or anti-GST immunoblotting. (D) His-PRMT1 incubated with GST-PRMT6 was subjected to Ni-NTA pulldown followed by anti-PRMT6 or anti-PRMT1 immunoblotting. (E) H1299 cells were co-transfected with vectors expressing YFPn-PRMT1 and YFPc-PRMT1, YFPn-PRMT6 and YFPc-PRMT6, YFPn-PRMT6 and YFPc-PRMT1, YFPn-PRMT1 and YFPc-MS2, or YFPn-PRMT6 and YFPc-MS2. YFP signal was analyzed by fluorescence microscopy. Scale bar, 25 μm. (F) Expression of transfected proteins in (E) was analyzed by anti-FLAG or anti-HA immunoblotting. (G) Extracts of H1299 or PRMT1 KO cells were subjected to anti-PRMT6 immunoprecipitation. The immunoprecipitates were analyzed by anti-PRMT1 or anti-PRMT6 immunoblotting.

PRMTs can function independently or cooperate with other PRMTs.^28^^,^^29^ To test the cooperation between PRMT6 and PRMT1 or PRMT4, we analyzed their interactions in transfected cells by coimmunoprecipitation assays and found that PRMT6 interacted with PRMT1, but not with PRMT4 (Figures 2A and 2B). Using recombinant proteins expressed in *E. coli,* we showed a direct protein-protein interaction between PRMT1 and PRMT6 (Figures 2C and 2D). To examine the interaction in living cells, we performed bimolecular fluorescence complementation (BiFC) assays,^32^ where two non-fluorescent fragments of a fluorescent protein form a fluorescent complex when fused to two proteins that interact with each other. We fused the N-terminal fragment of YFP (YFPn) or the C-terminal fragment of YFP (YFPc) to the N-terminus of PRMT1 or PRMT6 and detected the formation of PRMT1/PRMT1 homocomplex, PRMT6/PRMT6 homocomplex, and PRMT1/PRMT6 heterocomplex in BiFC assays (Figure 2E). All transfected proteins were expressed in the cells (Figure 2F). Finally, we demonstrated that endogenous PRMT1 and PRMT6 form a heterocomplex (Figure 2G).

### A peptide inhibitor disrupts the formation of PRMT1/PRMT6 heteromer

**Figure 3:**
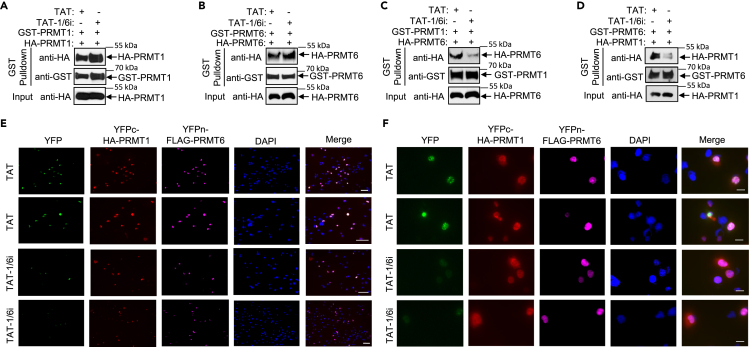
Development of a peptide inhibitor of PRMT1/PRMT6 heteromer (A) GST-PRMT1 incubated with cell extracts containing HA-PRMT1 in the presence of TAT (20 μM) or TAT-1/6i (20 μM) was subjected to GST pulldown followed by anti-HA or anti-GST immunoblotting. (B) GST-PRMT6 incubated with cell extracts containing HA-PRMT6 in the presence of TAT (20 μM) or TAT-1/6i (20 μM) was subjected to GST pulldown followed by anti-HA or anti-GST immunoblotting. (C) GST-PRMT1 incubated with cell extracts containing HA-PRMT6 in the presence of TAT (20 μM) or TAT-1/6i (20 μM) was subjected to GST pulldown followed by anti-HA or anti-GST immunoblotting. (D) GST-PRMT6 incubated with cell extracts containing HA-PRMT1 in the presence of TAT (20 μM) or TAT-1/6i (20 μM) was subjected to GST pulldown followed by anti-HA or anti-GST immunoblotting. See also Figures S3 and S4. (E and F) H1299 cells were co-transfected with vectors expressing YFPc-HA-PRMT1 and YFPn-FLAG-PRMT6 followed by treatment with TAT (200 μM) or TAT-1/6i (200 μM) for 24 h. Cells were subjected to anti-HA and anti-FLAG immunofluorescence. Signals were analyzed by fluorescence microscopy using 10× magnification (E, Scale bar, 100 μm) or 40× magnification (F, Scale bar, 20 μm).

To demonstrate the importance of the PRMT1/PRMT6 heteromer, we designed several peptides corresponding to amino acid sequences in the loops of PRMT1 or PRMT6 connecting *β*4 and *α*D involved in cofactor and substrate binding.^33^ These peptides were fused with a cell-penetrating peptide from the transactivator of transcription (TAT) of human immunodeficiency virus and tested for the ability to inhibit cell growth. We found that peptide #1, referred to as TAT-1/6i, reduced cell growth by 50% compared to the control peptide, TAT (Figure S3). We next established GST pulldown assays and detected interactions between GST-PRMT1 and HA-PRMT1, GST-PRMT6 and HA-PRMT6, GST-PRMT1 and HA-PRMT6, and GST-PRMT6 and HA-PRMT1 (Figures 3A–3D); no interactions were detected using GST as a control (Figure S4). Incubation with TAT-1/6i only disrupted the formation of the PRMT1/PRMT6 heteromer (Figures 3C and 3D), but not PRMT1/PRMT1 and PRMT6/PRMT6 homocomplexes (Figures 3A and 3B). We next examined whether TAT-1/6i disrupted PRMT1/PRMT6 heteromer in cells using BiFC assays. H1299 cells expressing both YFPc-HA-PRMT1 and YFPn-FLAG-PRMT6 were treated with TAT or TAT-1/6i and analyzed by immunofluorescence and YFP signals. While both YFPc-HA-PRMT1 and YFPn-FLAG-PRMT6 were expressed in the same transfected cells, treatment with TAT-1/6i resulted in a significant reduction in the number of YFP-positive cells (Figure 3E) and YFP signals (Figure 3F) compared to TAT treatment. These results demonstrate that TAT-1/6i disrupts PRMT1/PRMT6 heteromer.

### Disruption of the PRMT1/PRMT6 heteromer reduces cell proliferation

**Figure 4:**
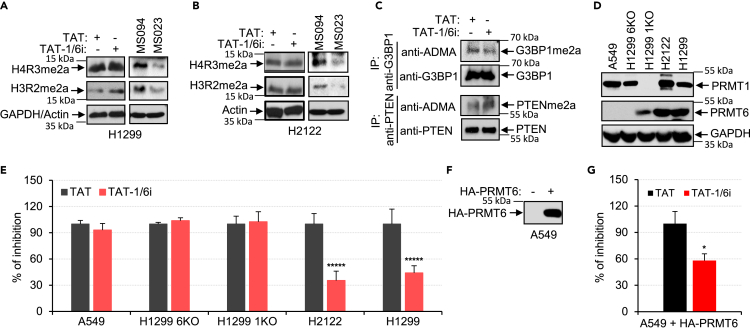
Disruption of PRMT1/PRMT6 heteromer reduces cell growth (A and B) Extracts of H1299 (A) or H2122 (B) cells treated with TAT (20 μM), TAT-1/6i (20 μM), MS094 (an inactive control for MS023, 100 nM), or MS023 (100 nM) for 48 h were analyzed by anti-H4R3me2a, anti-H3R2me2a, anti-GAPDH, or anti-Actin immunoblotting. (C) Extracts of H1299 cells treated with TAT (20 μM) or TAT-1/6i (20 μM) for 48 h were immunoprecipitated with anti-G3BP1 or anti-PTEN. The precipitates were analyzed by anti-ADMA, anti-G3BP1, or anti-PTEN immunoblotting. (D) Extracts of A549, H1299 PRMT6 KO, H1299 PRMT1 KO, H2122, or H1299 cells were analyzed by anti-PRMT1, anti-PRMT6, or anti-GAPDH immunoblotting. (E) Reduced growth of cells expressing both PRMT1 and PRMT6 by TAT-1/6i. Cells were treated with TAT (100 μM) or TAT-1/6i (100 μM) for 2 days and cell growth was analyzed. Cells treated with TAT was set at 100%. Data are mean (n = 3) ± SD.∗∗∗∗∗, p < 0.000005 (two-tailed t-test). (F) Extracts of A549 cells transfected with a vector expressing HA-PRMT6 were analyzed by anti-HA immunoblotting. (G) Transfected A549 cells in (F) were treated with TAT (250 μM) or TAT-1/6i (250 μM) for 4 days and cell growth was analyzed. Cells treated with TAT was set at 100%. Data are mean (n = 3) ± SD. ∗, p < 0.05 (two-tailed t-test).

To test the specificity and activity *in vitro*, we treated H1299 and H2122 cells with TAT-1/6i and analyzed arginine methylation on the substrates of PRMT1 or PRMT6, histone 4 (H4) or histone 3 (H3), respectively. No differences in the levels of asymmetrical dimethylation on H4R3 (H4R3me2a) and H3R2 (H3R2me2a) were detected by treatment with TAT-1/6i compared to TAT. In contrast, treatment with MS023, a broader type I PRMT inhibitor, reduced the levels of H4R3me2a and H3R2me2a (Figures 4A and 4B). Treatment with TAT-1/6i did not affect the arginine methylation of G3BP1, a substrate of PRMT1,^34^ and increased the arginine methylation of PTEN, a substrate of PRMT6^35^ (Figure 4C). These results indicate that arginine methylation activities of PRMT1 and PRMT6 toward their substrates were not affected by TAT-1/6i. We further validated the specificity of TAT-1/6i in cell proliferation using several NSCLC cell lines with varied expressions of PRMT1 and PRMT6 (Figure 4D). TAT-1/6i reduced the growth in cell lines that express both PRMT1 and PRMT6 (H1299 and H2122), but did not affect the growth in cell lines that lack either PRMT1 (H1299 PRMT1 KO) or PRMT6 (A549 and H1299 PRMT6 KO; Figure 4E). Furthermore, the expression of HA-PRMT6 in A549 cells rendered their sensitivity toward TAT-1/6i on cell proliferation (Figures 4F and 4G).

**Figure 5:**
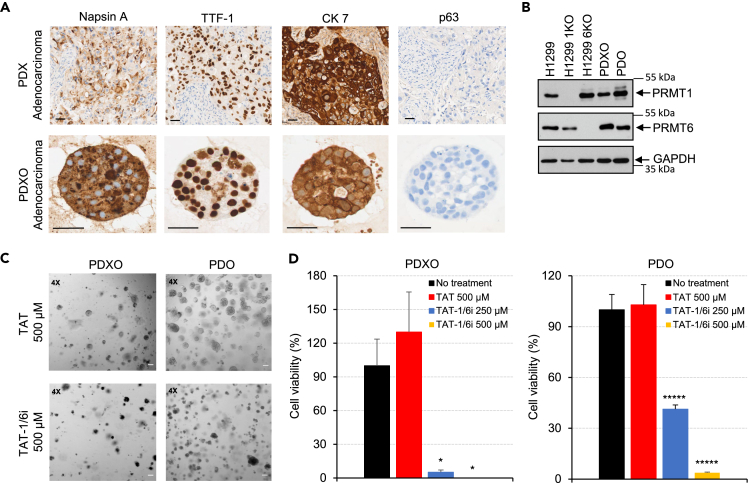
Disruption of PRMT1/PRMT6 heteromer inhibits the growth of LC PDXOs and PDOs (A) IHC-stained images of LC PDX tissue and PDXOs. Paraffin embedded sections of PDX and PDXOs were incubated with anti-Napsin A, anti-TTF-1, anti-CK7, or anti-p63 antibodies followed by incubation with secondary antibodies and visualized. PDX and PDXOs are positive for Napsin-A, TTF-1, and CK7 (LUAD markers) and negative for p63 (LUSC marker). Scale bar, 50 μm. (B) Extracts of H1299, H1299 1KO, H1299 6KO, PDXOs, and PDOs were analyzed by immunoblotting using anti-PRMT1, anti-PRMT6, or anti-GAPDH. (C and D) PDXOs and PDOs were treated with TAT (500 μM) or TAT-1/6i (250 or 500 μM) for 5 days. Bright-field images of treated PDXOs and PDOs were shown (C), and cell viability was analyzed (D). Scale bar, 25 μm. PDXOs and PDOs without the treatment was set at 100%. Data are mean (n = 3) ± SD. ∗, p < 0.05, ∗∗∗∗∗, p < 0.000005 (one-way ANOVA).

We then established PDXOs and PDOs from LUAD tissue to determine the effect of disruption of PRMT1/PRMT6 heteromer on cell growth. Figure 5A showed that the PDXOs retained the tumor organization and expression of LUAD markers, Napsin A, thyroid transcription factor 1 (TTF-1), and cytokeratin 7 (CK7), as compared to tissue from the original PDX. By contrast, no staining with anti-p63 (a LUSC marker) was detected. Both PRMT1 and PRMT6 were endogenously expressed in PDXOs and PDOs analyzed by immunoblotting (Figure 5B). Treatment of both PDXOs and PDOs with TAT-1/6i induced cell death determined by the microscopic observation of shrinkage of organoids (Figure 5C) and resulted in a significant decrease in cell viability (Figure 5D). We examined a possible mechanism of cell death and did not detect an induction of cleaved PARP and cleaved caspase 3, well-known apoptosis markers (data not shown), by the peptide suggesting that other pathways, e.g., autophagic cell death or necrosis, resulted in the observation in Figure 5C. By contrast, treatment with TAT had no effect. These results demonstrate that the disruption of the PRMT1/PRMT6 heteromer inhibits the growth of LC PDXOs and PDOs likely resulting from non-apoptotic cell death pathways.

### PRMT1/PRMT6 heteromer catalyzes the arginine methylation of ILF2

**Figure 6:**
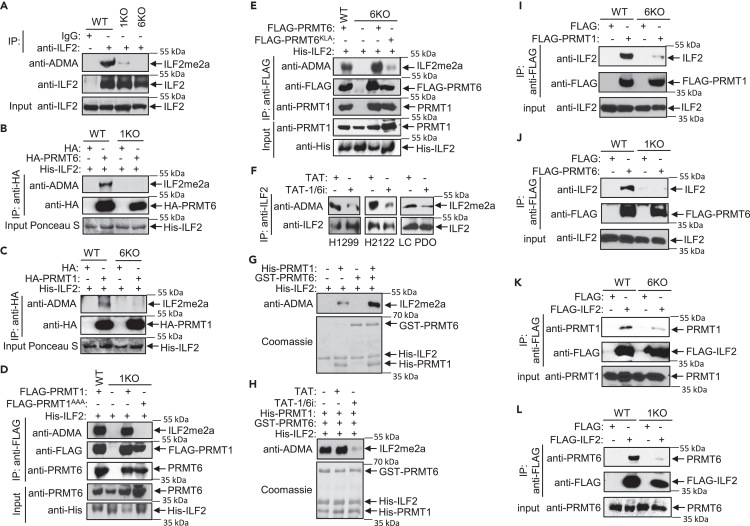
PRMT1/PRMT6 heteromer recruits and catalyzes the arginine methylation of ILF2 (A) Methylation of ILF2 in PRMT1 KO and PRMT6 KO cells is reduced. Extracts of H1299, PRMT1 KO, or PRMT 6KO cells were immunoprecipitated with anti-ILF2. The precipitates were analyzed by anti-ADMA or anti-ILF2 immunoblotting. (B) The ability of HA-PRMT6 to catalyze methylation on ILF2 is markedly reduced in the absence of PRMT1. HA-PRMT6 expressed in H1299 or PRMT1 KO cells was immunoprecipitated with anti-HA. The precipitates were incubated with His-ILF2 in methylation assays followed by anti-ADMA or anti-HA immunoblotting. (C) The ability of HA-PRMT1 to catalyze arginine methylation on ILF2 is markedly reduced in the absence of PRMT6. HA-PRMT1 expressed in H1299 or PRMT6 KO cells was immunoprecipitated with anti-HA. The precipitates were incubated with His-ILF2 in methylation assays followed by anti-ADMA or anti-HA immunoblotting. (D) Catalytic activity of PRMT1 is required for the methylation of ILF2. FLAG-PRMT1 expressed in H1299 or PRMT1 KO cells or FLAG-PRMT1^AAA^, a catalytically inactive PRMT1, expressed in PRMT1 KO cells were immunoprecipitated with anti-FLAG. The precipitates were incubated with His-ILF2 in methylation assays followed by anti-ADMA, anti-FLAG, or anti-PRMT6 immunoblotting. (E) Catalytic activity of PRMT6 is required for the methylation of ILF2. FLAG-PRMT6 expressed in H1299 or PRMT6 KO cells or FLAG-PRMT6^KLA^, a catalytically inactive PRMT6, expressed in PRMT6 KO cells were immunoprecipitated with anti-FLAG. The precipitates were incubated with His-ILF2 in methylation assays followed by anti-ADMA, anti-FLAG, or anti-PRMT1 immunoblotting. (F) Disruption of PRMT1/PRMT6 heteromer decreases the methylation of ILF2. H1299, H2122 cells, or LC PDOs were treated with TAT (50 μM for H1299 and H2122, 100 μM for LC PDOs) or TAT-1/6i (50 μM for H1299 and H2122, 100 μM for LC PDOs) for 24 h (H1299 and H2122) or 5 days (LC PDOs). Extracts were immunoprecipitated with anti-ILF2 followed by anti-ADMA or anti-ILF2 immunoblotting. (G) His-ILF2 was incubated with His-PRMT1, GST-PRMT6, or both His-PRMT1 and GST-PRMT6 in methylation assays followed by anti-ADMA immunoblotting. Coomassie blue staining of the membrane after immunoblotting is shown. (H) Both His-PRMT1 and GST-PRMT6 were incubated with TAT (500 nM) or TAT-1/6i (500 nM) in room temperature for 25 min, followed by the addition of His-ILF2 and methylation assays. The reactions were analyzed by anti-ADMA immunoblotting. Coomassie blue staining of the membrane after immunoblotting is shown. (I) Coprecipitation of ILF2 with FLAG-PRMT1 is reduced in the absence of PRMT6. FLAG-PRMT1 expressed in H1299 or PRMT6 KO cells was immunoprecipitated with anti-FLAG followed by anti-ILF2 or anti-FLAG immunoblotting. (J) Coprecipitation of ILF2 with FLAG-PRMT6 is reduced in the absence of PRMT1. FLAG-PRMT6 expressed in H1299 or PRMT1 KO cells was immunoprecipitated with anti-FLAG followed by anti-ILF2 or anti-FLAG immunoblotting. (K) Coprecipitation of PRMT1 with FLAG-ILF2 is reduced in the absence of PRMT6. FLAG-ILF2 expressed in H1299 or PRMT6 KO cells was immunoprecipitated with anti-FLAG followed by anti-PRMT1 or anti-FLAG immunoblotting. (L) Coprecipitation of PRMT6 with FLAG-ILF2 is reduced in the absence of PRMT1. FLAG-ILF2 expressed in H1299 or PRMT1 KO cells was immunoprecipitated with anti-FLAG followed by anti-PRMT6 or anti-FLAG immunoblotting.

We previously demonstrated that the association of ILF2 with PRMT6 potentiated lung tumor progression.^36^ We therefore examined whether the PRMT1/PRMT6 heteromer could catalyze the arginine methylation of ILF2. ILF2 was immunoprecipitated from H1299, PRMT1 KO, or PRMT6 KO cells followed by immunoblotting using anti-asymmetric dimethylarginine (ADMA) antibodies. ILF2 methylation was significantly reduced in PRMT1 KO and PRMT6 KO cells compared to the parental cells (Figure 6A). We next performed *in vitro* methylation assays to analyze the abilities of ectopically expressed HA-tagged PRMT1 or PRMT6 to catalyze ILF2 methylation. HA-PRMT1 or HA-PRMT6 immunoprecipitated from H1299 cells efficiently introduced ADMA on ILF2. By contrast, HA-PRMT1 immunoprecipitated from PRMT6 KO cells or HA-PRMT6 immunoprecipitated from PRMT1 KO cells failed to methylate ILF2 (Figures 6B and 6C). To examine the role of catalytic activities of PRMT1 and PRMT6 in the methylation of ILF2, we expressed a PRMT1 mutant, PRMT1^AAA,^^37^ or a PRMT6 mutant, PRMT6^KLA,^^38^ which lack catalytic activity, in PRMT1 KO or PRMT6 KO cells. Arginine methylation of ILF2 by PRMT1 mutant or PRMT6 was significantly reduced (Figures 6D and 6E) indicating that the enzymatic activities of both PRMT1 and PRMT6 are required. Furthermore, treatment of NSCLC cells and LC PDOs with TAT-1/6i reduced arginine methylation of ILF2 (Figure 6F) indicating that PRMT1/PRMT6 heteromer is responsible for ILF2 methylation. We further examined the arginine methylation of ILF2 by PRMT1 and PRMT6 using recombinant proteins expressed in *E. coli*. Although PRMT1 alone, but not PRMT6 alone, could methylate ILF2, the presence of both PRMT1 and PRMT6 strongly methylated ILF2 (Figure 6G). More importantly, the arginine methylation of ILF2 introduced by PRMT1/PRMT6 heteromer was significantly decreased by the treatment of TAT-1/6i (Figure 6H).

We next examined the recruitment of ILF2 into the PRMT1/PRMT6 heteromer by co-immunoprecipitation assays. While ILF2 was efficiently coprecipitated with FLAG-PRMT1 or FLAG-PRMT6 in H1299 cells, the coprecipitation was dramatically reduced in the absence of PRMT6 or PRMT1, respectively (Figures 6I and 6J). Similarly, coprecipitation of PRMT1 or PRMT6 with FLAG-ILF2 was significantly reduced in the absence of PRMT6 or PRMT1, respectively (Figures 6K and 6L). These results demonstrate the ability of the PRMT1/PRMT6 heteromer to recruit/recognize and therefore catalyze arginine methylation on a new class of substrates, e.g., ILF2.

### PRMT1/PRMT6 heteromer positively regulates ILF2 expression

**Figure 7:**
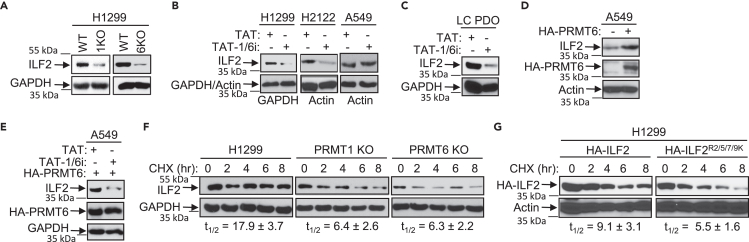
PRMT1/PRMT6 heteromer positively regulates ILF2 expression (A) Reduced ILF2 expression in PRMT1 KO and PRMT6 KO cells. Cell extracts were subjected to anti-ILF2 or anti-GAPDH immunoblotting. (B) Reduced ILF2 expression by TAT-1/6i. Extracts of H1299, H2122, or A549 cells treated with TAT (20 μM) or TAT-1/6i (20 μM) for 48 h were analyzed by immunoblotting using anti-ILF2, anti-GAPDH, or anti-Actin. (C) Extracts of LC PDOs treated with TAT (100 μM) or TAT-1/6i (100 μM) for 5 days were analyzed by immunoblotting using anti-ILF2 or anti-GAPDH. (D) Extracts of A549 cells transfected with a control vector or a vector expressing HA-PRMT6 were analyzed by anti-ILF2, anti-PRMT6, or anti-Actin immunoblotting. (E) A549 cells transfected with a vector expressing HA-PRMT6 were treated with control TAT (20 μM) or TAT-1/6i (20 μM) for 48 h. Cell extracts were subjected to immunoblotting with anti-ILF2, anti-PRMT6, or anti-GAPDH. (F) Decreased ILF2 protein stability in PRMT1 KO and PRMT6 KO cells. H1299, PRMT1 KO, or PRMT6 KO cells were treated with cycloheximide (50 μg/mL). Levels of ILF2 and GAPDH were analyzed 0, 2, 4, 6, and 8 h after treatment by immunoblotting. Half-life (t_1/2_) of ILF2 was calculated and presented as mean ± SD (n = 3). (G) Decreased stability of HA-ILF2^R2/5/7/9K^. HA-tagged wild-type ILF2 or ILF2^R2/5/7/9K^ was expressed in H1299 cells. Their stabilities were measured as in (D). Half-life of ILF2 was calculated and presented as mean ± SD (n = 3). See also Figures S5–S7.

We examined the effect of the PRMT1/PRMT6 heteromer on ILF2 expression and detected a significant reduction in ILF2 levels in PRMT1 KO and PRMT6 KO cells (Figure 7A). Disruption of PRMT1/PRMT6 heteromer resulted in a reduction of ILF2 expression in H1299 and H2122 cells, but not in A549 cells lacking PRMT6 expression (Figure 7B), and in LC PDOs (Figure 7C). Expression of HA-PRMT6 in A549 cells increased ILF2 expression (Figure 7D) and rendered their sensitivity to TAT-1/6i-induced ILF2 inhibition (Figure 7E).

We next tested whether the change in ILF2 expression resulted from protein stability. ILF2 stability was significantly decreased in PRMT1 KO and PRMT6 KO cells compared to H1299 cells (Figure 7F). To determine the effect of arginine methylation on ILF2 expression, we identified methylation sites by mass spectrometry. ILF2 containing five potential RG sites at the N-terminus (Figure S5A) was incubated with both His-PRMT1 and GST-PRMT6 in a methylation assay. We identified a peptide with dimethylated R2, a peptide with dimethylated R5, a peptide with dimethylated R7, and a peptide containing R9 with a monomethyl group (Figures S5B–S5F). We were unable to find a peptide containing R16. These results suggest that R2, R5, and R7 are dimethylated, and R9 is likely dimethylated as we detected a monomethyl group which is an intermediate product of type I PRMTs. Although we could not detect whether all four Rs are dimethylated within a peptide, our results suggested that they are likely simultaneously dimethylated or randomly dimethylated by PRMT1/PRMT6 heteromer. To confirm this, we mutated all four arginines to lysines and examined the effect on methylation by PRMT1/PRMT6 heteromer. ILF2 carrying mutations (ILF2^R2/5/7/9K^) blocked the methylation by the heteromer (Figures S5G and S5H) and exhibited a shorter half-life compared to wild-type ILF2 (Figure 7G). Together, our results demonstrate that arginine methylation by PRMT1/PRMT6 heteromer positively regulates ILF2 expression resulting from increased protein stability.

### Higher ILF2 expression is detected in lung adenocarcinoma of Black/AA men

Previous studies have shown that the increased expression of ILF2 promotes cell proliferation and is associated with worse overall survival in patients with NSCLC.^39^^,^^40^ Using TCGA datasets and Kaplan-Meier Plotter, we found the upregulation of ILF2 in LUAD and an association of higher ILF2 expression with poorer survival (Figures S6A and S6B). We did not observe higher ILF2 mRNA levels in LUAD of Black/AA men versus NHW men (Figure S6C). However, higher ILF2 protein levels were detected in fresh frozen LUAD tissue of Black/AA men versus NHW men (Figures S6D and S6E). These results further support the findings that PRMT1/PRMT6 heteromer positively drives ILF2 expression in LUAD of Black/AA men via control of protein stability.

### ILF2 is essential for cell proliferation

To investigate whether the effect of PRMT1/PRMT6 heteromer on cell proliferation acts through ILF2, we re-expressed HA-PRMT1 in PRMT1 KO cells and FLAG-PRMT6 in PRMT6 KO cells. Re-expression of PRMT1 or PRMT6 in the respective knockout cell lines restored ILF2 levels (Figures S7A and S7B) and cell proliferation (Figure S7C). We next examined the role of ILF2 in cell growth. Knockdown of ILF2 by siRNAs reduced cell growth in PRMT1 KO, PRMT1 KO with the re-expression of PRMT1, PRMT6 KO, and PRMT6 KO with the re-expression of PRMT6 (Figures S7D–S7F). Taken together, our results suggest that the increased expression of ILF2 by the PRMT1/PRMT6 heteromer promotes cell proliferation and likely contributes to lung cancer health disparities in Black/AA men.

### Arginine methylation of ILF2 by PRMT1/PRMT6 heteromer counteracts ubiquitination by CRL4^CRBN^ E3 ligase

**Figure 8:**
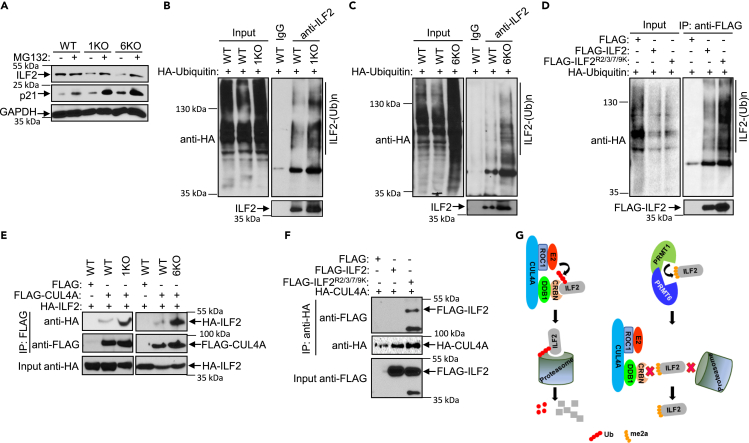
PRMT1/PRMT6-mediated methylation counteracts the ubiquitination of ILF2 (A) H1299, PRMT1 KO, or PRMT6 KO cells were treated with MG132 (5 μM) for 8 h. Levels of ILF2, p21, and GAPDH were analyzed by immunoblotting. (B and C) H1299 or PRMT1 KO cells (B) and H1299 or PRMT6 KO cells (C) were transfected with a vector expressing HA-ubiquitin. Cell extracts were subjected to anti-ILF2 immunoprecipitation followed by anti-HA or anti-ILF2 immunoblotting. (D) H1299 cells were co-transfected with a control vector or vectors expressing FLAG-ILF2 or FLAG-ILF2^R2/5/7/9K^ and a vector expressing HA-ubiquitin. Cell extracts were subjected to anti-FLAG immunoprecipitation followed by anti-HA or anti-FLAG immunoblotting. (E) H1299, PRMT1 KO, or PRMT6 KO cells were co-transfected with vectors expressing FLAG-CUL4A and HA-ILF2. Cell extracts were subjected to anti-FLAG immunoprecipitation followed by anti-FLAG or anti-HA immunoblotting. (F) H1299 cells were co-transfected with a control vector or vectors expressing FLAG-ILF2 or FLAG-ILF2^R2/5/7/9K^ and a vector expressing HA-CUL4A. Cell extracts were subjected to anti-HA immunoprecipitation followed by anti-HA or anti-FLAG immunoblotting. (G) Illustration of CRL4^CRBN^ complex and PRMT1/PRMT6 heteromer in the regulation of ILF2 expression.

To determine the underlying mechanism of ILF2 regulation by PRMT1/PRMT6 heteromer, we treated H1299, PRMT1 KO, and PRMT6 KO cells with MG132, a proteasome inhibitor, and observed that ILF2 levels were restored in PRMT1 KO and PRMT6 KO cells. Meanwhile, the levels of ILF2 were not affected in H1299 cells (Figure 8A). MG132 treatment also increased the levels of p21 subjected to degradation by proteasomes. These results suggest that ILF2 is undergoing significant proteasome-mediated degradation in the absence of PRMT1 or PRMT6. We next measured the levels of ubiquitination of ILF2 in H1299, PRMT1 KO, and PRMT6 KO cells. Ubiquitination of ILF2 was increased in both PRMT1 KO and PRMT6 KO cells compared to H1299 cells (Figures 8B and 8C). Additionally, ubiquitination of mutant ILF2 (ILF2^R2/5//7/9K^) was increased compared to wild-type ILF2 (Figure 8D), indicating that the methylation of ILF2 prevents the ubiquitination of ILF2. It has been shown that the CUL4-RBX1-DDB1-CRBN (CRL4^CRBN^) E3 ubiquitin ligase complex regulates ILF2 turnover.^41^ Thus, we examined the effect of the PRMT1/PRMT6 heteromer on the recruitment of ILF2 into CRL4^CRBN^ complex. The levels of ILF2 coprecipitated with CUL4A was increased in the absence of PRMT1 or PRMT6 (Figure 8E). In addition, the association of ILF2^R2/5/7/9K^ with CUL4A was significantly increased compared to wild-type ILF2 (Figure 8F). Altogether, our results demonstrate that ILF2 is degraded by a ubiquitination-mediated process and arginine methylation by PRMT1/PRMT6 heteromer counteracts the action of CRL4^CRBN^ complex on ILF2 (Figure 8G).

## Discussion

Here, we reported that, among the members of type I PRMT family, levels of PRMT1, PRMT4, and PRMT6 were elevated in NSCLC, and high expression was associated with poorer patient survival. Furthermore, PRMT6 expression was significantly increased in LUAD tissue of Black/AA men compared to NHW men. We also demonstrated that PRMT1 and PRMT6 form a heteromer essential for cell proliferation. Together, these results and our previous findings that mice with the lung-specific expression of PRMT6 develop more lung tumors^36^ strongly suggest that elevated PRMT6 expression in cooperation with PRMT1 drives lung cancer development and likely contributes to health disparities in Black/AA men. However, we cannot completely rule out that PRMT6 alone via the formation of homodimers contributes to the observed disparities in Black/AA men.

It has been shown that PRMT1 is co-purified with PRMT6^29^ and PRMT6 is a substrate of PRMT1, in which enzymatic activity of PRMT6 is decreased by methylation.^30^ However, the significance of this interaction contributing to cell proliferation and cancer development is still unknown. We demonstrated that PRMT1/PRMT6 heteromer played a critical role in cell proliferation and perhaps lung cancer development. Although the formation of the PRMT1/PRMT6 heteromer is via direct protein-protein interactions, the topology of the complex is still unknown. While PRMT1 and PRMT6 can form a heterodimer, we speculate that they can also form a heterotetramer containing a PRMT1 homodimer and a PRMT6 homodimer and/or an oligomer containing more than one PRMT1 homodimer and one PRMT6 homodimer. This is based on our findings that a competitive peptide corresponding to the loop region of PRMT1 connecting the β4 and αD, which is not essential for the formation of PRMT homodimer mediated between the dimerization arm (α4-6 helices) and helices αY/Z and α1/2 of the Rossmann domain,^42^ disrupts the formation of PRMT1/PRMT6 heteromer.

Type I PRMTs recognize and catalyze the arginine methylation of various substrates, including histones and non-histone proteins.^12^^,^^13^ We demonstrated that the PRMT1/PRMT6 heteromer recruits and catalyzes the methylation of a novel substrate, e.g., ILF2, which is weakly recognized by either PRMT1 or PRMT6 homodimers (Figure 6). Although the details of substrate recognition by the PRMT1/PRMT6 heteromer are currently unknown, our data implicate that the heteromer catalyzes arginine methylation on a battery of proteins playing critical roles in a variety of cellular processes. Therefore, it is of interest to identify these substrates and characterize their functions in lung cancer development and their role in health disparities.

Our results indicate that the enzymatic activities of both PRMT1 and PRMT6 are essential for catalyzing the arginine methylation of ILF2. The data showed that no methylation of ILF2 by the complex containing PRMT6 and catalytically inactive PRMT1 was detected (Figure 6D). In contrast, some methylation by the complex containing PRMT1 and catalytically inactive PRMT6 was detected (Figure 6E). Using recombinant PRMT1 and PRMT6, we showed that PRMT1 alone could methylate ILF2, but not PRMT6. However, working together as a heteromer yielded a synergistic affect toward increased ILF2 methylation (Figure 6G). Currently, the exact mechanism by which PRMT1/PRMT6 heteromer catalyzes ILF2 methylation is unclear. It is possible that the methylation of ILF2 by PRMT1 is further activated by PRMT6-mediated methylation of PRMT1 although Cao et al. showed that PRMT6 was methylated by PRMT1.^30^ Alternatively, the methylation of ILF2 at specific arginine sites within the RG motifs by PRMT1 could render further methylation at distinct arginine sites by PRMT6.

It has been previously shown that ILF2 interacts with CRBN, a known substrate receptor, the domains required for the interaction need further investigation. We speculate that the interaction between the N-terminus of ILF2 and CRBN is required for recruitment into the CRL4^CRBN^ complex to undergo ubiquitination. We postulate that methylation by PRMT1/PRMT6 heteromer will prevent the interaction between ILF2 and CRBN, thereby inhibiting the ubiquitination-mediated degradation of ILF2 (Figure 8G). Although the dysregulation of ILF2 is involved in multiple cancer types,^24^^,^^25^^,^^26^ its role in lung cancer is unclear. Our results showed higher ILF2 levels in lung tumor samples compared to the matched uninvolved lungs^36^ and in lung cancer tissues of Black/AA men compared to NHW men (Figure S6D). We further demonstrated that PRMT1/PRMT6 heteromer’s effect on cell proliferation acted through ILF2 (Figure S7), suggesting a positive effect of ILF2 on cell growth. Thus, it is of interest to determine the role of ILF2 in lung cancer development and in mediating the function of the PRMT1/PRMT6 heteromer *in vivo.*

To demonstrate the proof of principle for the targeting of the PRMT1/PRMT6 heteromer in lung cancer, we showed that disrupting the complex reduced cell viability in LC PDOs from a Caucasian male (PDXO) and a Black/AA female (PDO). Since higher PRMT6 expression, thus higher PRMT1/PRMT6 heteromer, is found in LUAD of Black/AA men, targeting the complex will be most beneficial for this cohort. It is also of interest to compare the effectiveness of the competitive peptide in LUAD PDOs derived from Black/AA men versus NHW men.

In summary, our study identified a unique role of the PRMT1/PRMT6 heteromer in driving lung cancer development which likely contributes to cancer health disparities in Black/AA men. Targeting the PRMT1/PRMT6 heteromer could create a new class of PRMT inhibitors in treating lung cancer to overcome the current limitations of PRMT1 or PRMT6 inhibitors, thereby opening up new horizons for efficacious PRMT1/PRMT6-targeted agents that are expected to eliminate lung cancer health disparities.

### Limitations of the study

In this study, we found that higher PRMT6 expression in LUAD of Black/AA men versus NHW men using a limited sample number. This observation needs to be further validated using a larger cohort. The higher PRMT6 expression correlated with worse outcomes in Black/AA men versus NHW men should be also further investigated. Although our data indicate that disrupting PRMT1/PRMT6 heteromer reduces cell proliferation using NSCLC cell lines, it remains uncertain whether this is also occurring *in vivo*. Thus, the next step is to utilize genetically engineered mouse models to demonstrate the role of PRMT1/PRMT6 heteromer in lung cancer. While we demonstrate that PRMT1/PRMT6 heteromer recognizes and catalyzes arginine methylation on a new class of substrates, we still do not know the list of substrates and the mechanism of recognition and catalyzation of the substrates. Furthermore, the underlying mechanism by which the heteromer drives lung cancer and causes worse outcomes in Black/AA men with lung cancer is still unclear. Our study using NSCLC cell lines indicates that ILF2 plays a critical in cell proliferation and is likely a downstream effector of PRMT1/PRMT6 heteromer. However, the *in vivo* role of ILF2 in driving lung cancer and whether the function of PRMT1/PRMT6 heteromer is mediated via ILF2 still await further investigation. We demonstrate that the methylation of ILF2 by PRMT1/PRMT6 heteromer counteracts the action of ubiquitination by CRL4^CRBN^ complex. However, the mechanism by which methylation affects ubiquitination and whether CRL4^CRBN^ directly catalyzes the ubiquitination of ILF2 are not provided in the study.

## STAR★Methods

### Key resources table

**Table:** 

REAGENT or RESOURCE	SOURCE	IDENTIFIER
**Antibodies**		

6∗His, His-Tag Monoclonal antibody	Proteintech	Cat# 66005-1; RRID: AB_11232599
Alexa Fluor™ Plus 647- conjugated secondary antibodies	Invitrogen	Cat# A32733; RRID: AB_2633282
Anti-Actin antibody	Sigma-Aldrich	Cat# A2066; RRID: AB_476693
anti-CK7	Proteintech	Cat# 15539-1-AP; RRID: AB_2249769
Anti-dimethyl-Histone H3 (Arg2), clone 20.2	Sigma-Aldrich	Cat# 04-808; RRID: AB_1587125
Anti-Histone H4R3me2a antibody	Sigma-Aldrich	Cat# SAB4300868
Anti-ILF2 antibody	Sigma-Aldrich	Cat# HPA007484; RRID: AB_1079137
anti-Napsin A	Lecia Biosystems	Cat# NAPSIN-L-CE
Anti-p21 antibody	Abcam	Cat# ab7960; RRID: AB_306174
anti-p63	Cell Signaling Technology	Cat# 39692; RRID: AB_2799159
Anti-rabbit IgG, HRP-linked Antibody	Cell Signaling Technology	Cat# 7074; RRID: AB_2099233
anti-TTF-1	Lecia Biosystems	Cat# TTF-1-L-CE
Asymmetric Di-Methyl Arginine Motif [adme-R] MultiMab™ Rabbit mAb mix	Cell Signaling Technology	Cat# 13522; RRID: AB_2665370
Cy3-conjugated secondary antibodies	Jackson ImmunoResearch	Cat# 115-165-003; RRID: AB_2338680
DYKDDDDK Tag (D6W5B) Rabbit mAb	Cell Signaling Technology	Cat# 14793; RRID: AB_2572291
GAPDH (D16H11) XP® Rabbit mAb	Cell Signaling Technology	Cat# 5174; RRID: AB_10622025
Goat anti-Mouse IgG (H + L) Secondary Antibody, HRP	Invitrogen	Cat# 31430; RRID: AB_228307
GST Tag Polyclonal antibody	Proteintech	Cat# 10000-0-AP; RRID: AB_11042316
HA-Tag (6E2) Mouse mAb	Cell Signaling Technology	Cat# 2367; RRID: AB_10691311
Monoclonal ANTI-FLAG® M2 antibody	Sigma-Aldrich	Cat# F1804; RRID: AB_262044
PRMT1 (A33) Antibody	Cell Signaling Technology	Cat# 2449; RRID: AB_2237696
PRMT4/CARM1 (C31G9) Rabbit mAb #3379	Cell Signaling Technology	Cat# 3379; RRID: AB_2068433
PRMT6 (D5A2N) Rabbit mAb	Cell Signaling Technology	Cat# 14641; RRID: AB_2798552
PTEN Antibody	Cell Signaling Technology	Cat# 9552; RRID: AB_10694066
Purified Mouse Anti-Human G3BP	BD Transduction Laboratories	Cat# 611126; RRID: AB_398437
Rabbit anti-NF45 Antibody	Bethyl Laboratories	Cat# A303-147A; RRID: AB_10895652
Rabbit anti-PRMT1 Antibody	Bethyl Laboratories	Cat# A300-722A; RRID: AB_533421
Rabbit anti-PRMT6 Antibody	Bethyl Laboratories	Cat# A300-929A; RRID: AB_2237733
Recombinant Anti-ILF2/NF45 antibody [EPR10694(B)]	Abcam	Cat# ab154169
VeriBlot for IP Detection Reagent (HRP)	Abcam	Cat# ab131366; RRID: AB_2892718
Vinculin Antibody	Cell Signaling Technology	Cat# 4650; RRID: AB_10559207
β-Actin (8H10D10) Mouse mAb	Cell Signaling Technology	Cat# 3700; RRID: AB_2242334

**Bacterial and virus strains**		

BL21 (DE3) Competent E. coli	ThermoFisher	Cat# EC0114
Dh5-Alpha Competent E. coli	Zymo	Cat# T3007
JM109 Competent E. coli	Promega	Cat# L2001
Origami™ 2(DE3) Competent Cells	Novagen	Cat# 71345

**Biological samples**		

(Uninvolved) lung tumor frozen tissue	Tissue and Data Acquisition and Analysis Core-VCU	N/A
Lung tumor specimen to generate patient-derived organoid (PDO)	Division of Surgical Oncology, VCU School of Medicine	N/A
Patient-derived xenograft to generate PDX-organoid (PDXO)	VCU Massey Cancer Center-Cancer Mouse Models Core	N/A

**Chemicals, peptides, and recombinant proteins**		

MG132	SIGMA	Cat# M8699
MS0293	Dr. Vedadi (Canada)	N/A
MS0294	Dr. Vedadi (Canada)	N/A
S-adenosylmethionine (SAM)	NEB	Cat# B9003S
TAT Control (YGRKKRQRRR) and TAT-YESMLNTVL (YGRKKRQRRR-YESMLNTVL)	LifeProtein	Cat# LT118590(1) and Cat# LT116025(6)

**Critical commercial assays**		

Bond Polymer Refine Detection kit	Leica Biosystems	Cat# DS9800
Brilliant II SYBR® Green QPCR Master Mix	Agilent	Cat# 600828
CellTiter-Glo 3D Cell Viability Assay	Promega	Cat# G9681

**Deposited data**		

Mass Spectrometry data	Database: MassIVE Repository	Dataset identifiers: MSV000093674

**Experimental models: Cell lines**		

Human: A549 (human NSCLC)	ATCC	Cat# CCL-185; CVCL_0023
Human: H1299 (human NSCLC)	ATCC	Cat# CRL-5803; RRID: CVCL_0060
Human: H2122 (human NSCLC)	ATCC	Cat# CRL-5985; RRID: CVCL_1531
Human: HEK293 (human kidney)	ATCC	Cat# CRL-1573; RRID: CVCL_0045

**Oligonucleotides**		

Control siRNA	QIAGEN	Cat# 1027281
Primer: hPRMT1 Reverse CATGGACTCGTAGAAGAG	Eurofins	N/A
Primers: hPRMT1 Forward CTCTGATTATGCGGTGAA	Eurofins	N/A
Primers: hPRMT6 Forward GGAGCTGGAGGCCGGAGTGGG	Invitrogen	N/A
Primers: hPRMT6 Reverse GCGTGATCTCTCCTGAAACGT	Invitrogen	N/A
sgRNA targeting sequence: hPRMT1: first exon ACACAGUACUCAGUUGAUA	Synthesized at University of Illinois at Chicago - Genomic Services	N/A
sgRNA targeting sequence: hPRMT6: first exon GAAAAGAAAGCTTGAGTCGG	Synthesized at University of Illinois at Chicago - Genomic Services	N/A
siRNA: hILF2	Dharmacon	Cat# M-017599-00-0005
siRNA: hPRMT1	Dharmacon	Cat# M-010102-01-0005
siRNA: hPRMT6	Dharmacon	Cat# M-007773-00-0005

**Recombinant DNA**		

Plasmid: pCDNA3.1-FLAG-CUL4A	Wang et al.^43^	N/A
Plasmid: pCDNA3.1-FLAG-hPRMT1(PRMT1^AAA^, PRMT6, PRMT6^KLA^, ILF2, ILF2^R2/5/7/9K^), pCDNA3.1-Flag	This paper	N/A
Plasmid: pCDNA-HA-CUL4 A	Wang et al.^43^	N/A
Plasmid: pCDNA-HA-PRMT4	Addgene	Cat# 81118; RRID: Addgene_81118
Plasmid: pCDNA-HA-ubiquitination	Wang et al.^43^	N/A
Plasmid: pCMV-HA-hPRMT1 (PRMT6, ILF2, ILF2^R2/5/7/9K^), pCMV-HA	Avasarala et al.^36^	N/A
Plasmid: Pet28a^+^-PRMT1 (ILF2, ILF2^R2/5/7/9K^), Pet28a^+^	Avasarala et al.^36^ and this paper	N/A
Plasmid: pGEX-hPRMT6 (hPRMT1), pGEX4T1	Avasarala et al.^36^	N/A
Plasmid: pMDG	Addgene	Cat# 187440; RRID: Addgene_187440
Plasmid: psPAX2	Addgene	Cat# 12260; RRID: Addgene_12260
Plasmid: YFPc-HA-PRMT1 (PRMT6, MS2BP)	Stöhr et al.^44^	N/A
Plasmid: YFPn-FLAG-PRMT1 (PRMT6)	Stöhr et al^44^	N/A
pLV[Exp]-Hygro-CMV>HA/hPRMT1	VectorBuilder	Cat# VB220111-1256qgv
pLV[Exp]-Puro-CMV>EGFP	VectorBuilder	Cat# VB900088-2219pdm
pLV[Exp]-Puro-CMV>FLAG/hPRMT6	VectorBuilder.	Cat# VB220111-1254ztj

**Software and algorithms**		

Adobe Photoshop	This paper (figures)	https://www.adobe.com/
BioRender	This paper (graphical Abstract)	https://biorender.com
Excel	This paper (analysis, figures)	https://www.office.com
GraphPad Prism 9	This paper (figures)	https://www.graphpad.com/
ImageJ	This paper (analysis, quantification)	https://imagej.net/ij/

**Other**		

ANTI-FLAG® M2 Affinity Gel	Millipore	Cat# A2220
Bond Primary antibody diluent	Leica Biosystem	Cat# AR9352
Cultrex Ultimatrix Reduced Growth Factor Basement Membrane Extract	R&D Systems	Cat# BME001-05
Epitope retrieval buffer 1	Leica Biosystem	Cat# RE7113-CE
Epitope retrieval buffer 2	Leica Biosystem	Cat# RE7119-CE
HisPur™ Ni-NTA Resin	Thermo Scientific	Cat# 88221
Histogel	Thermo Scientific	Cat# HG-4000-012
jetOPTIMUS®	Polyplus	Cat# 101000006
Jurkat Apoptosis Cell Extracts (etoposide)	Cell Signaling Technology	Cat# 2043
Lipofectamine™ 2000 Transfection Reagent	Invitrogen	Cat# 11668019
Lipofectamine™ Transfection Reagent	Invitrogen	Cat# 18324012
N-2 Max Media Supplement (100X)	R&D Systems	Cat# AR009
N-21 Max Media Supplement (50X)	R&D Systems	Cat# AR008
Pierce™ Glutathione Superflow Agarose	Thermo Scientific	Cat# 25236
Protein G Resin	GenScript	Cat# L00209
Recombinant Human EGF Protein	R&D Systems	Cat# AFL236-200
Recombinant Human FGF basic Protein	R&D Systems	Cat# 234-FSE-025/CF
VECTASHIELD® Antifade Mounting Medium	Vector Laboratories, Inc.	Cat# H1000
Y-27632 dihydrochloride	R&D Systems	Cat# 1254

### Resource availability

#### Lead contact

Further information and requests for resources and reagents should be directed to and will be fulfilled by the lead contact, Robert A. Winn (Robert.Winn@vcuhealth.org).

#### Materials availability

All the materials generated in this study are available from the lead contact upon request.

#### Data and code availability

• Proteomics data have been deposited at MassIVE Repository and are publicly available as of the date of publication. Accession numbers are listed in the key resources table. All data reported in this paper will be shared by the lead contact upon request. This paper does not report original code.
• Any additional information required to reanalyze the data reported in this paper is available from the lead contact upon request.

### Experimental model and study participant details

#### Lung cancer specimens

De-identified frozen lung tumor tissue samples were obtained from The Lung Cancer Biospecimen Resource Network (LCBRN) at the University of Virginia, and from the Tissue and Data Acquisition and Analysis Core (TDAAC) at Virginia Commonwealth University. Race was self-identified. For the generation of lung cancer (LC) patient-derived organoids (PDOs), informed consent was obtained and lung tumor tissue collection was carried out under VCU Internal Review Board approved protocol, HM20021072. For the generation of LC PDXOs, organoids were established from patient-derived xenografts (PDXs) in collaboration with the Cancer Mouse Models Shared Resource at VCU Massey Cancer Center.

#### Cell lines and cell culture

NSCLC cell lines, A549, H1299, and H2122, were obtained from the American Type Culture Collection (ATCC). Cell lines were cultured in RPMI 1640 medium supplemented with 10% FBS and 1% penicillin-streptomycin in a humidified CO_2_ (5%) incubator at 37°C. All cell lines in this study were passaged less than 10 times and routinely tested for Mycoplasma contamination.

### Method details

#### LC patient-derived organoids (PDOs)

Single-cell suspension from lung tumor tissues or PDXs (∼4 mm^3^) were obtained by enzymatic dissociation in 5 mL of Advanced DMEM/F12 media supplemented with collagenase type II (1 mg/mL), DNase I (0.025 mg/mL, final concentration), Y-27632 dihydrochloride (10 μM final concentration, R&D Systems, 1254), penicillin-streptomycin (1%, final concentration) and amphotericin B (2.5 μg/mL, final concentration) for 2 h in a 37°C rotating oven. Cell suspension was strained through a 70-micron cell strainer and centrifuged at 600 rcf for 3 min at room temperature. Cell count was conducted using a Nexcelcom Cellometer and cells were seeded in a ratio of 100,000 cells per 40 μL dome of Cultrex Ultimatrix Reduced Growth Factor Basement Membrane Extract (BME, R&D Systems, BME001-005) in a 6-well non-treated tissue culture plate and cultured for 7–14 days with supplemented growth medium (Advanced DMEM/F12 containing 1% penicillin-streptomycin, 20 ng/mL of rhFGF-basic protein (R&D Systems, 234-FSE-025/CF), 50 ng/mL of hEGF protein (R&D Systems, AFL236-200), N2-Max Media Supplement (100X, R&D Systems, AR009), N21-Max Media Supplement (50X, R&D Systems, AR008), 10μM of Y-27632 dihydrochloride (R&D Systems) to allow for organoid formation.^45^ Medium was replaced every 3–4 days and organoids were passed at a 1:3 ratio upon reaching confluency. For passaging, organoids were harvested in 2 mL wash media [Advanced DMEM/F12 supplemented with 10% Fetal Bovine Serum – Embryonic Stem Cell Qualified (R&D Systems)] and then centrifuged at 600 rcf for 3 min at room temperature. Organoids were dissociated in 2 mL TrypLE (Gibco) and incubated in a 37°C water bath for 45 min and centrifuged at 600 rcf for 3 min at room temperature. The pellet was incubated with DNase I (0.025 mg/mL final concentration) in 1 mL wash media for 5 min at room temperature and centrifuged at 600 rcf for 3 min. The pellet was resuspended in BME at a 1:3 ratio and reseeded for new growth. Organoids that reached passage 3 were processed for protein extraction or cell viability assay.

#### 3D organoid cell viability assay

Organoids in Basement Membrane Extract (BME) were dissociated enzymatically and cells were counted and re-seeded in BME (7,500 cells/5 μL) in a black 96-well tissue culture plate. The cells were cultured in supplemented growth media for 7 days or until the size of organoids increased by more than 2-fold. Organoids were then treated with control TAT or TAT-1/6i peptides for 5 days.^46^ Cell viability was analyzed by CellTiter-Glo 3D Cell Viability Assay (Promega, G9681) per manufacturer’s instruction.

#### Histology and immunohistochemistry (IHC)

LC PDX tissue and PDXOs were fixed in 10% neutral buffered formalin in which PDXOs were further suspended in Histogel (Thermo Scientific, HG-4000-012), followed by processing, paraffin embedding, and sectioning. IHC was performed by the VCU Tissue and Data Acquisition and Analysis Core with the Lecia Bond RX autostainer using either heat-induced epitope retrieval buffer 2 (Leica Biosystem, RE7119-CE) for anti-Napsin A, anti-TTF-1, and anti-CK7 staining, or epitope retrieval buffer 1 (Leica Biosystem, RE7113-CE) for anti-p63 staining, for 20 min. The antibodies diluted in Bond Primary antibody diluent (Leica Biosystem, AR9352) were added and incubated for an additional 15 min (anti-CK7) or 45 min (anti-Napsin A, anti-TTF-1, and anti-p63). Antibodies were as follows: anti-Napsin A (1:200 dilution, Lecia Biosystems, NAPSIN-L-CE), anti-TTF-1 (1:600 dilution, Lecia Biosystems, TTF-1-L-CE), anti-CK7 (1:200 dilution, Proteintech, 15539-1-AP), and anti-p63 (1:600 dilution, Cell Signaling, 39692). Primary antibody incubation was followed by incubation with secondary antibodies for 8 min and HRP detection with DAB for 10 min using the Bond Polymer Refine Detection kit (Leica Biosystems). Stained slides were then imaged on the Vectra Phenoimager HT (formerly Vector Polaris, Akoya Biosciences).

#### Plasmids, lentiviral vectors, and reagents

Plasmids expressing FLAG- or HA-tagged human PRMT1, PRMT6, and ILF2 were generated by subcloning the corresponding cDNAs into pCDNA3.1 or pCMV-HA vectors, respectively. Vectors expressing catalytically inactive PRMT1 (PRMT1^AAA^), PRMT6 (PRMT6^KLA^), and ILF2 with arginine to lysine mutations (ILF2^R2/5/7/9K^) were generated by PCR-mediated site-directed mutagenesis and confirmed by DNA sequencing. To express PRMT1, PRMT6, and ILF2 in *E. coli,* the corresponding cDNAs were either subcloned into pGEX-4T1 or pET-28 a (+) vectors. A plasmid expressing HA-tagged PRMT4 was purchased from Addgene (#81118). Plasmids expressing HA-tagged CUL4A and ubiquitin were previously described^43^ and provided by Dr. Hengbin Wang (Massey Cancer Center, VCU). Plasmids expressing YFPn-FLAG-PRMT1, YFPn-FLAG-PRMT6, YFPc-HA-PRMT1, and YFPc-HA-PRMT6 were generated by subcloning the corresponding cDNAs into vectors expressing YFPn or YFPc^44^ provided by Dr. Jose Cuzeva (University of Madrid, Spain). A plasmid expressing YFPc-HA-MS2 was kindly provided by Dr. Jose Cuzeva. Lentiviral vectors expressing GFP, HA-tagged PRMT1, and FLAG-tagged PRMT6 were purchased from VectorBuilder. A control siRNA (#1027281) was purchased from Qiagen. siRNA targeting ILF2 (M-017599-00-0005) was purchased from (Dharmacon). TAT and TAT-1/6i peptides were synthesized and purchased from LifeProtein LLC.

#### The cancer genome atlas (TCGA) data analysis

LUAD and LUSC data were downloaded from TCGA database using R/Bioconductor package ‘TCGAbiolinks’.^47^ Genomic Data Commons (GDC) API search parameters include: Gene expression quantification, RNA-seq, Illumina HiSeq, and results. Clinical data were queried using ‘GDCquery_clinic’. The raw RNA-seq data were downloaded using ‘GDCdownload’ function in ‘TCGAbiolinks’ package and normalized using ‘TCGAanalyze_Normalization’ implemented in R/Bioconductor package ‘EDASeq’.^48^ The normalized data were further filtered using ‘TCGAanalyze_Filtering’. Differential expression analysis was performed using ‘TCGAanalyze_DEA’ function inherited from R/Bioconductor package ‘edgeR’.

#### Gene editing of PRMT1 and PRMT6

Guide RNAs targeting the first exons of PRMT1 (CATGATGCAGTTCGCGGCCT) and PRMT6 (GAAAAGAAAGCTTGAGTCGG) were used to generate PRMT1 knockout (KO) and PRMT6 KO.^36^ H1299 cells were transfected with the guide plasmids using Lipofectamine 2000 reagent (Invitrogen). 48 h after transfection, cells were trypsinized, diluted to a concentration of 1 cell/100μL, seeded into 96-well plates (1 cell/well), and grown for 3 weeks. Single clones were isolated and assayed by immunoblot analysis for PRMT1 and PRMT6 expression. Multiple clones that showed complete loss of PRMT6 expression were selected for downstream analysis.

#### Lentiviral production and transduction

HEK293T cells (8 × 10^6^ cells) were seeded onto a 100-mm culture dish. Cells were transfected 16 h later with a lentiviral vector expressing a respective gene (12 μg), pMD.G (2 μg), and psPAX2 (8 μg) using Lipofectamine 2000. Medium was replaced with fresh growth medium (7 mL) containing sodium butyrate (10 mM) 16 h later. Medium containing lentivirus was collected 24 h later, centrifuged, filtered using a 0.45 μm syringe filter, and saved at −80°C. Cells were further incubated in fresh growth medium (7 mL) containing sodium butyrate (5 mM), and medium was collected 24 h later, filtered, and saved at −80°C. To transduce cells with lentiviral vectors expressing HA-PRMT1 or FLAG-PRMT6, PRMT1 KO or PRMT6 KO cells (3 × 10^5^ cells/well) were seeded onto a 6-well dish and incubated with 1 mL of growth medium containing lentivirus and polybrene (8 μg/mL) for 16 h. The transduced cells were selected for 7 days in the presence of hygromycin B (400 μg/mL) for HA-PRMT1 or puromycin (0.75–1 μg/mL) for FLAG-PRMT6.

#### Protein expression and purification in *E. coli*

Single colony of transformed BL21 (for GST fusion) or OrigamiTM2 (DE3) (for His fusion) expressing His- or GST-hPRMT1, GST-hPRMT6, or His-ILF2 was inoculated to 2 mL of growth medium and incubated overnight at 37°C with shaking. The next day, the culture was diluted 1:50 to fresh medium and continued to grow for 2 h followed by the addition of 0.5 mM of isopropyl-β-D-thiogalactoside (IPTG). The culture was incubated at 25°C with shaking for additional 6 h and harvested. The pellets were re-suspended in 10 mL of lysis buffer [125 mM Tris-HCl (pH8.0), 150 mM sodium chloride, 1 μM PMSF, 250 μg/mL lysozyme, and 0.5% Triton X-100]. Genomic DNA was shared by sonication followed by centrifugation. Protein purification was carried out per Thermo Scientific procedures using Pierce Glutathione Superflow Agarose for GST fusion proteins or HisPur Ni-NTA Resin for His fusion proteins.

#### Transient transfection

Cells were seeded at 80% confluency in cell culture plates and transfected using Lipofectamine (Invitrogen, 18324012), Lipofectamine 2000 (Invitrogen, 11668019) or jetOPTIMUS reagent (Polyplus, 101000006). The cells were harvested and processed after 24–48 h.

#### Cell proliferation assays

Cell proliferation was measured by Sulforhodamine B (SRB) assays.^49^ Briefly, cells (1,500-2,500 cells/well) were cultured in a 96-well plate and harvested at different time points. Cells were fixed with 10% trichloroacetic acid for 30 min at 4°C and subjected to SRB staining. Briefly, 70 μL of 0.4% (w/v) SRB in 1% acetic acid solution were added to the cells, followed by incubation at room temperature for 20 min. The cells were washed with 1% acetic acid to remove unbound SRB. Bound SRB was solubilized with 200 μL of 10 mM unbuffered Tris solution. Absorbance was read at 492 nm subtracting the background measurement at 620 nm. Cell growth rate was calculated by normalizing the readings of each time point with 0 time point, which was set at 1, to control for plating of equal number of cells among different treatments.

#### Biomolecular fluorescence complementation assays

Transfected cells (10,000–30,000 cells/well) were seeded in a chamber with tissue culture-treated glass slide (Falcon, 354118). For immunofluorescence analysis, cells were fixed with 4% paraformaldehyde for 20 mins and then permeabilized with 1X PBS containing 0.1% Triton X-100. Cells were incubated with anti-FLAG (1:400 dilution; Cell Signaling, 14793), and anti-HA (1:100 dilution; Cell Signaling, 2367) overnight at 4°C. Alexa Fluor Plus 647- conjugated secondary antibodies (1:400 dilution; Invitrogen, A32733) and Cy3-conjugated secondary antibodies (1:1000 dilution; Jackson ImmunoResearch, 115-165-003) were added followed by incubation at 37°C for 30 mins. Cells were rinsed several times with 1X PBS, stained with DAPI (1 μg/mL; Invitrogen, 62247), and mounted with Vectashield Antifade mounting medium (Vector Laboratories, Inc., H1000). Image acquisition was performed using Olympus Microscope IX71 or BZ-X810 All-in-One Fluorescent Microscopes.

#### Immunoprecipitation, pulldown, and immunoblot analyses

Extracts from NSCLC cells or fresh frozen lung tissue samples were prepared in NP-40 lysis buffer [150 mM NaCl, 50 mM Tris-HCl (pH 8), and 1% NP-40]. Proteins were either immunoprecipitated with specific antibodies using G resin (GenScript, L00209) or anti-FLAG M2 affinity gel (Sigma, A2220), or pulled down using Pierce Glutathione Superflow Agarose (ThermoFisher, 25236) or HisPur Ni-NTA Resin (ThermoFisher, 88221) at 4°C overnight. The precipitates were extensively washed with NP-40 lysis buffer, resolved on SDS-PAGE gels, and analyzed by immunoblotting.^49^

#### *In vitro* methylation assays

*In vitro* methylation assay was conducted using either bacterially produced recombinant proteins or immunoprecipitates.^50^ Briefly, H1299 cells were transfected transiently with HA (FLAG)–PRMTs (10 μg) in 100 mm culture dishes. Post 48 h of transfection, cells were lysed in NP40 lysis buffer, followed by the immunoprecipitation methods from this study. The bacterial recombinant proteins were generated according to the protocols in this study. Subsequently, immunoprecipitates, containing bound PRMTs or recombinant PRMTs, were incubated with His-ILF2 or His-ILF2^R2/5/7/9K^ (1–2 μg) in a reaction buffer composed of 50 mM Tris (pH 8.5), 20 mM KCl, 10 mM MgCl_2_, 1 mM β-mercaptoethanol, 100 mM sucrose, and S-adenosyl-L-methionine (final concentration: 500 μM) at 30°C for 1–1.5 h. Reactions were stopped by adding 6X SDS sample buffer, followed by denaturation through incubation at 95°C for 10 min. Samples were resolved via SDS-PAGE and subjected to immunoblotting using the anti-ADMA antibody (Cell Signaling, 13522).

#### RNA isolation and quantitative RT-PCR

Total RNA from lung tissues was extracted using Trizol reagent (Invitrogen). 1 μg of total RNA was reverse transcribed using random primers. Real-time PCR was performed using the Brilliant II SYBR Green QPCR Master Mix (Agilent, 600828) and the Bio-Rad CFX96 qPCR detection system. The primers used for PCR experiments are listed in the key resources table.

#### Mass spectrometry

His-ILF2 was incubated with both His-PRMT1 and GST-PRMT6 in a methylation assay. The reaction was resolved by SDS-PAGE followed by Coomassie blue staining. The band corresponding to ILF2 was excised from the gel, cut into equal size cubes (approximately 1 mm^3^), and transferred to a siliconized tube. The gel pieces were washed with 80% acetonitrile (CAN) for 10 min, followed by a H_2_O wash for 10 min. Gel pieces were dehydrated with 50% ACN for 5 min and vacuum centrifuged for 20 min. Samples were rehydrated with 20 mM DTT and incubated for 1 h at 56°C, followed by incubation with 60 mM iodoacetimide for 45 min in the dark. The pieces were dehydrated with 50% ACN for 10 min and vacuum centrifuged for 20 min. To rehydrate the gel cubes, samples were incubated for 10 min in 20 μL of 0.1% Protease Max (PM) trypsin (12.5 ng/mL) in 100 mM ammonium bicarbonate (AB). The gel pieces were then overlaid with 30 μL of 0.1% PM in 100 mM AB and allowed to digest for 2 h at 37°C. The digests were collected into fresh siliconized tubes, centrifuged at 14,000 rpm for 5 min, transferred to a fresh tube, and stored for subsequent mass spectrometry analysis. For LC-MS/MS, samples were analyzed using a Q Exactive HFX (Thermo Scientific) coupled to an Easy nLC 1200 (Thermo Scientific). The LC-MS system consisted of a Thermo Electron Q-Exactive HF-X mass spectrometer with an Easyspray Ion source connected to an Acclaim PepMap 75 μm × 2 cm nanoviper C18 3 μm × 100 Å pre-column in series with an Acclaim PepMap RSLC 75 μm × 50 cm C18 2 μm bead size (Thermo Scientific). Peptides were injected onto the column and then eluted from the column with an 80% ACN and 0.1% formic acid gradient at a flow rate of 0.3 μL/min over 1 h. The nano-spray ion source was operated at 1.9 kV. The digests were analyzed using the rapid switching capability of the instrument thus acquiring a full scan mass spectrum to determine peptide molecular weights followed by product spectra (25 High Energy C- trap Dissociation HCD spectra). This mode of analysis produces approximately 50,000 MS/MS spectra of ions ranging in abundance over several orders of magnitude. Not all MS/MS spectra are derived from peptides. The data were analyzed by database searching using the Sequest HT search algorithm with semi-tryptic enzyme digest using a custom database containing ILF2 downloaded from Swiss Prot. The data were processed with Proteome Discoverer 3.0. Searches including post-translational modifications (PTMs) of oxidation of methionine and carbamidomethyl cysteine. The number of missed cleavages for a tryptic search was set to 7. Modifications per peptide were set to 8. PTM search was set for methyl and di-methyl.

### Quantification and Statistical analysis

Data were collected from at least two independent, replicate experiments. All data are presented as the mean ± SD. Statistical significance (p value) was calculated by two-tailed t-test or one-way ANOVA (∗, p < 0.05; ∗∗, p < 0.005; ∗∗∗, p < 0.0005; ∗∗∗∗, p < 0.00005; ∗∗∗∗∗, p < 0.000005).
