# Supplementary material for: Cooperation between PRMT1 and PRMT6 drives lung cancer health disparities among Black/African American men
Source: iScience. 2024 Jan 11;27(2):108858. doi: 10.1016/j.isci.2024.108858 (PMC10830871; doi:10.1016/j.isci.2024.108858)
Supplement: Document S1. Figures S1–S7 [file mmc1.pdf]

iScience, Volume *27*

Supplemental information

Cooperation between PRMT1 and PRMT6 drives lung cancer health disparities among Black/African American men

Pei-Ying Wu, Michelle Van Scoyk, Stephanie S. McHale, Chu-Fang Chou, Gregory Riddick, Kamran Farouq, Bin Hu, Vita Kraskauskiene, Jennifer Koblinski, Charles Lyons, Arjun Rijal, Vignesh Vudatha, Dongyu Zhang, Jose G. Trevino, Rachit D. Shah, Patrick Nana-Sinkam, Yong Huang, Shwu-Fan Ma, Imre Noth, Chanita Hughes-Halbert, Victoria L. Seewaldt, Ching-Yi Chen, and Robert A. Winn

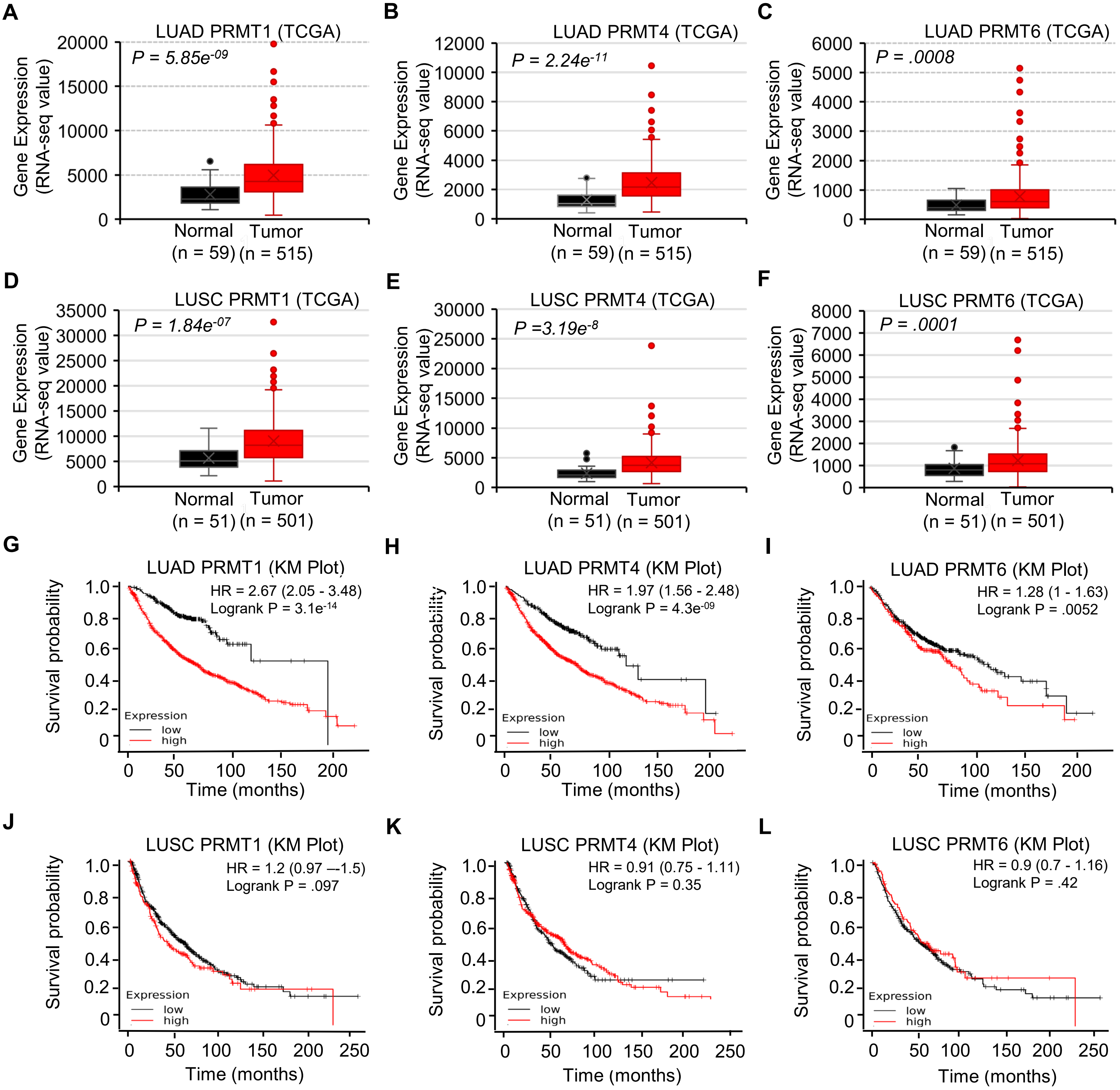

Figure S1. PRMT1, PRMT4, and PRMT6 are upregulated in NSCLC, Related to Figure 1. (A-C) Elevated expression of PRMT1, PRMT4, and PRMT6 in LUAD analyzed from TCGA datasets. Data are presented by Box and Whisker Plots, and P values were calculated by two-tailed t-test. (D-F) Elevated expression of PRMT1, PRMT4, and PRMT6 in LUSC analyzed from TCGA datasets. Data are presented by Box and Whisker Plots, and P values were calculated by two-tailed t-test. (G-L) Correlation of higher expression of PRMT1, PRMT4, and PRMT6 with poorer survival in patients with LUAD (G-I), but not patients with LUSC (J-L) analyzed by Kaplan-Meier Plotter.

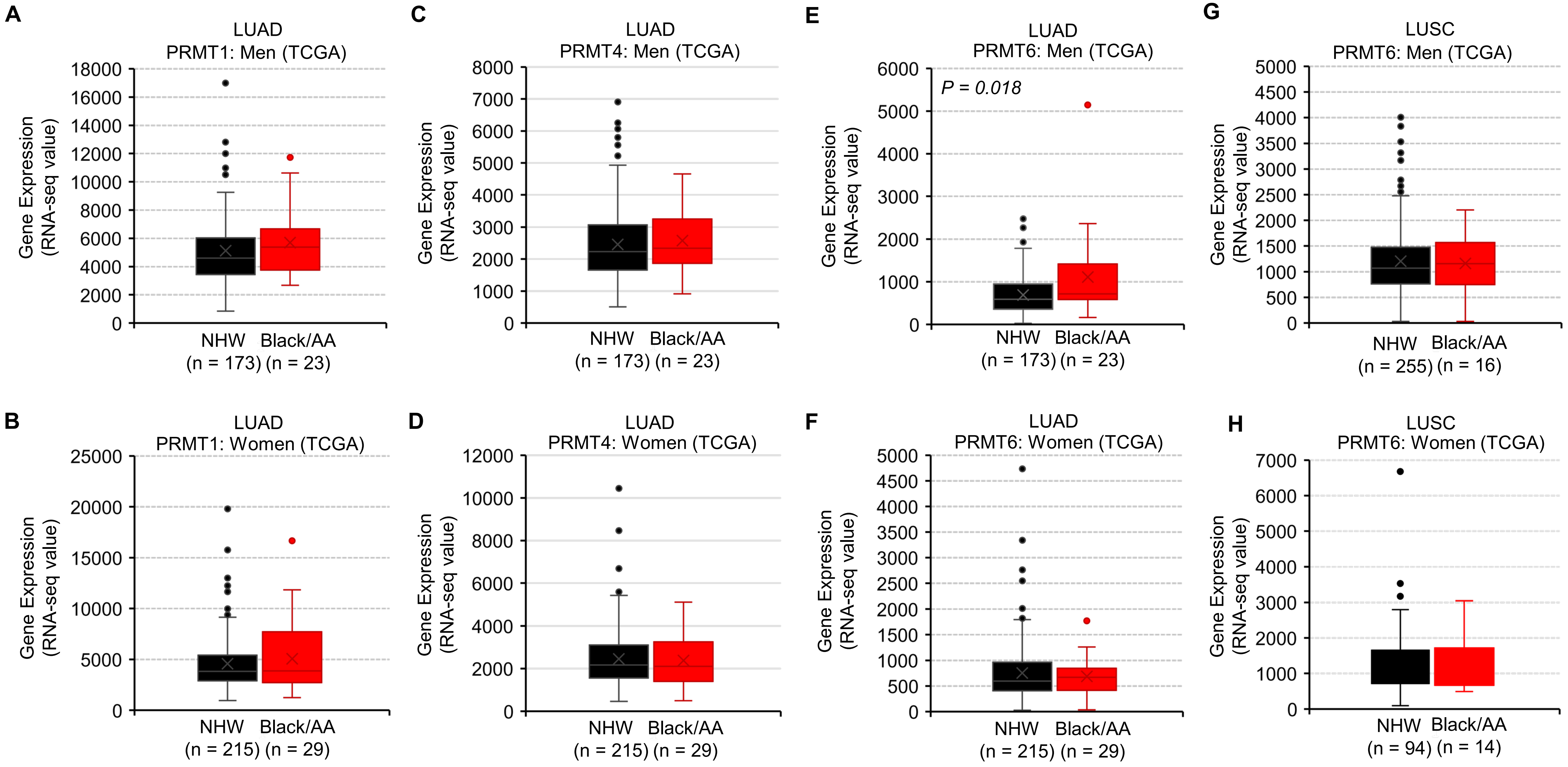

Figure S2. Higher PRMT6 expression is observed in LUAD of Black/AA men compared to NHW men analyzed from TCGA datasets, Related to Figure 1. (A-D) No differences in expression of PRMT1 and PRMT4 between Black/AA and NHW. (E and F) Higher PRMT6 levels in LUAD of Black/AA men compared to NHW men (E), but no such difference between Black/AA women and NHW women (F). (G and H) No difference in expression of PRMT6 in LUSC between Black/AA and NHW. Data are presented by Box and Whisker Plots, and P values were calculated by two-tailed t-test.

A

Amino acid sequences within the loop connecting β4 and αD:

PRMT1   EWMGYCLFYESMLNTVL
PRMT6   EWMGYGLLHESMLSSVL

B

Control: YGRKKRRQRRR
Peptide #1:   YGRKKRRQRRRYESMLNTVL
Peptide #2:   YGRKKRRQRRREWMGYGLLHE
Peptide #3:   YGRKKRRQRRRGLLHESMLSS

C

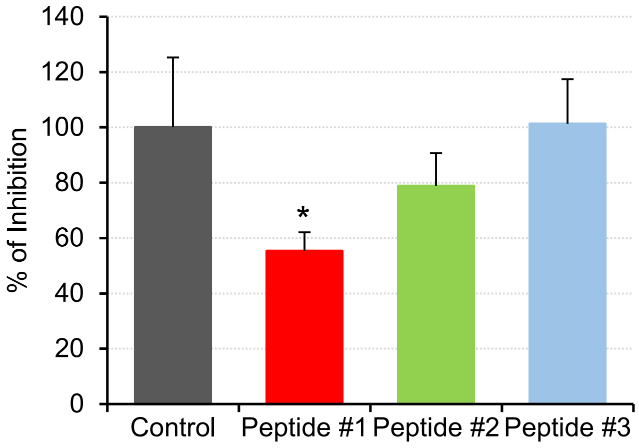

Figure S3. Identification of a peptide inhibiting cell growth, Related to Figure 3. (A) Amino acid sequences within the loops of human PRMT1 and PRMT6 connecting β4 and αD are shown. (B) The amino acids from the loop regions of PRMT1 or PRMT6 denoted in red, blue, or underlined are fused to TAT (control). (C) Cells were treated with the peptides indicated in (B) for 2 days and cell growth was analyzed. Cells treated with the control peptide was set at 100%. Data are mean (n = 3) ± SD. *, $P < 0.05$ (one-way ANOVA).

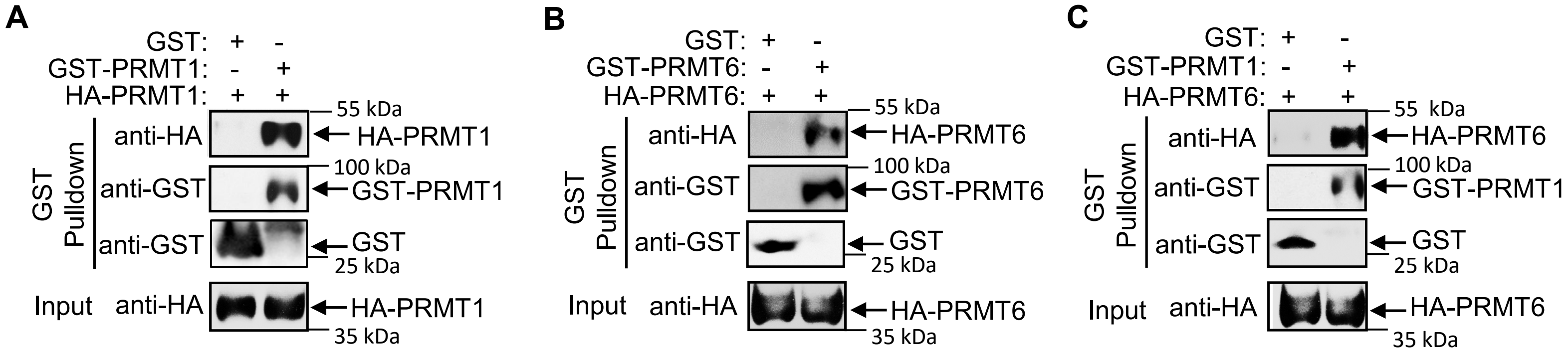

Figure S4. Complex formation between PRMT1 and PRMT1, PRMT6 and PRMT6, and PRMT1 and PRMT6, Related to Figure 3. (A) GST or GST-PRMT1 incubated with cell extracts containing HA-PRMT1 was subjected to GST pulldown followed by anti-HA or anti-GST immunoblotting. (B) GST or GST-PRMT6 incubated with cell extracts containing HA-PRMT6 was subjected to GST pulldown followed by anti-HA or anti-GST immunoblotting. (C) GST or GST-PRMT1 incubated with cell extracts containing HA-PRMT6 was subjected to GST pulldown followed by anti-HA or anti-GST immunoblotting.

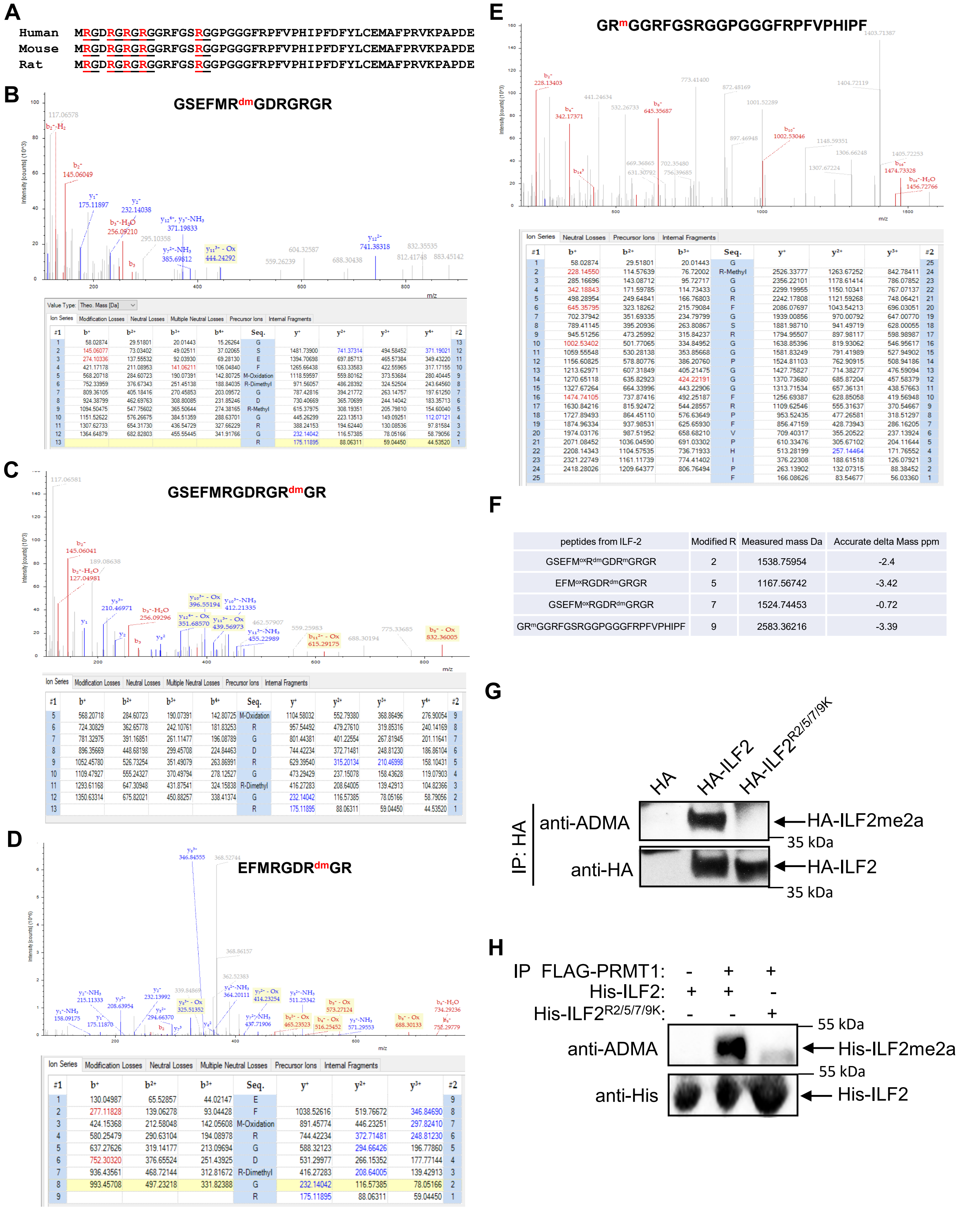

Figure S5. Identification of methylation sites by mass spectrometry, Related to Figure 7. (A) The N-terminus of ILF2 from human, mouse, and rat is shown with RG motifs underlined. (B-E) MS_2 scan and fragment map for the b and y ions identifying GSEFM^{ox}R^{dm}GDR^{m}GRGR (ox: oxidation, dm: dimethyl and m: methyl) with dimethylated R2 (B), EFM^{ox}RGDR^{dm}GRGR with dimethylated R5 (C), GSEFM^{ox}RGDRGR^{dm}GR with dimethylated R7 (D),and GR^{m}GGRFGSRGGPGGGFRPFVPHIPF with methylated R9 (E). (F) A table showing peptide sequences and modifications of R2, R5, R7, and R9 with the measured mass of each peptide and the delta mass of the measured mass vs. the calculated accurate mass in ppm (G) HA-ILF2 or HA-ILF2$^{R2/5/7/9K}$ was expressed in H1299 cells and immunoprecipitated with anti-HA. The precipitates were analyzed by anti-ADMA or anti-HA immunoblotting. (H) FLAG-PRMT1 was immunoprecipitated from H1299 cell extracts and subjected to methylation assays using His-ILF2 or His-ILF2$^{R2/5/7/9K}$ as a substrate. Methylation of ILF2 was detected by anti-ADMA immunoblotting.

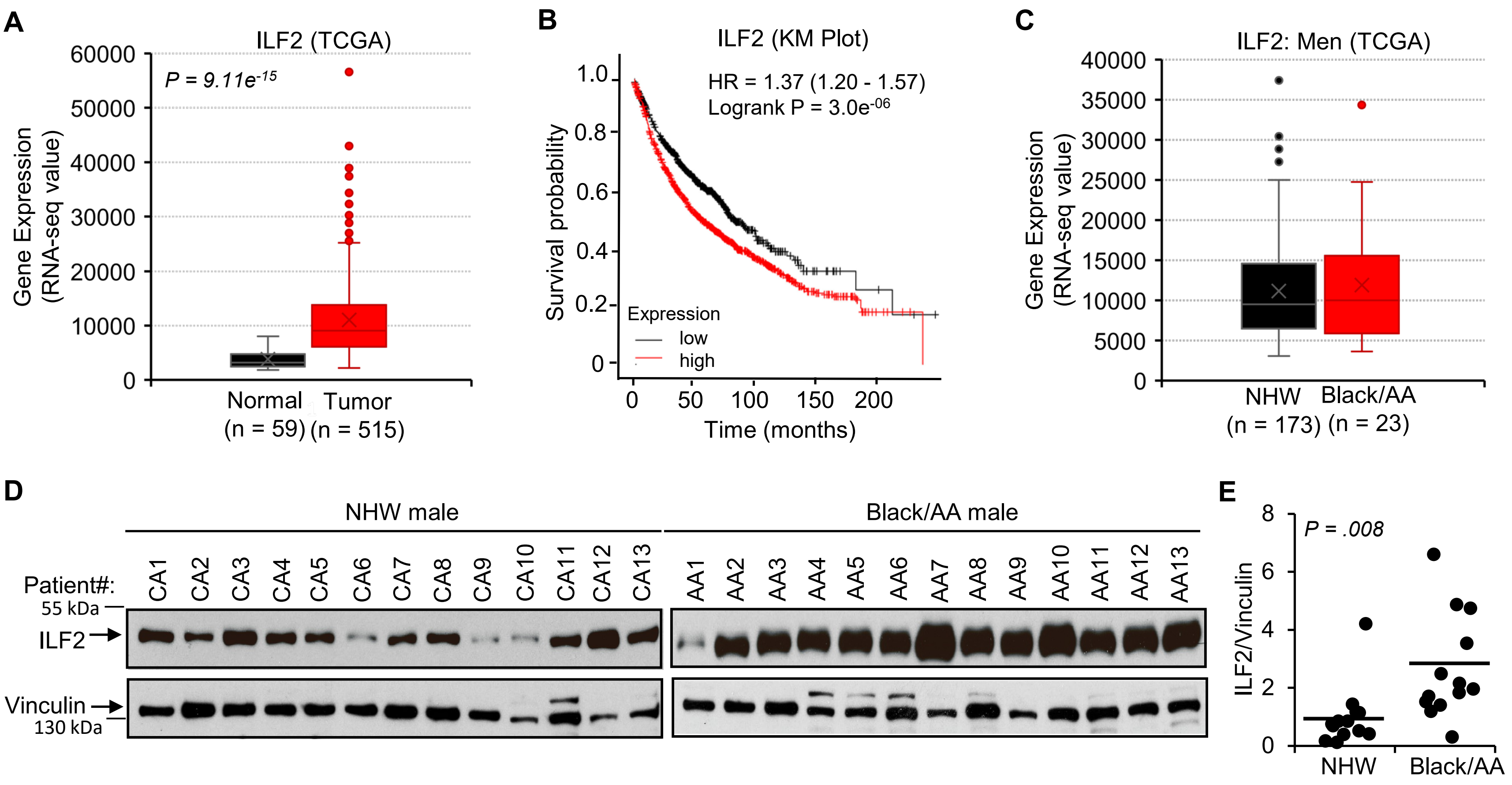

Figure S6. ILF2 expression is increased in LUAD, and higher ILF2 expression is observed in LUAD of Black/AA men compared to NHW men, Related to Figure 7. (A) ILF2 mRNA levels in normal and LUAD analyzed using TCGA datasets. Data are presented by Box and Whisker Plots, and P values were calculated by two-tailed t-test. (B) Association of ILF2 expression with patient survival was analyzed using Kaplan-Meier Plotter. (C) ILF2 mRNA levels between Black/AA men and NHW men analyzed using TCGA LUAD datasets. Data are presented by Box and Whisker Plots. (D) Higher ILF2 expression in LUAD tissue of Black/AA men compared to NHW men. The anti-vinculin image shown is the same one shown in Figure 1C. (E) Quantitation of signals in (D) is shown. Bars indicate the mean of all samples (two-tailed t-test).

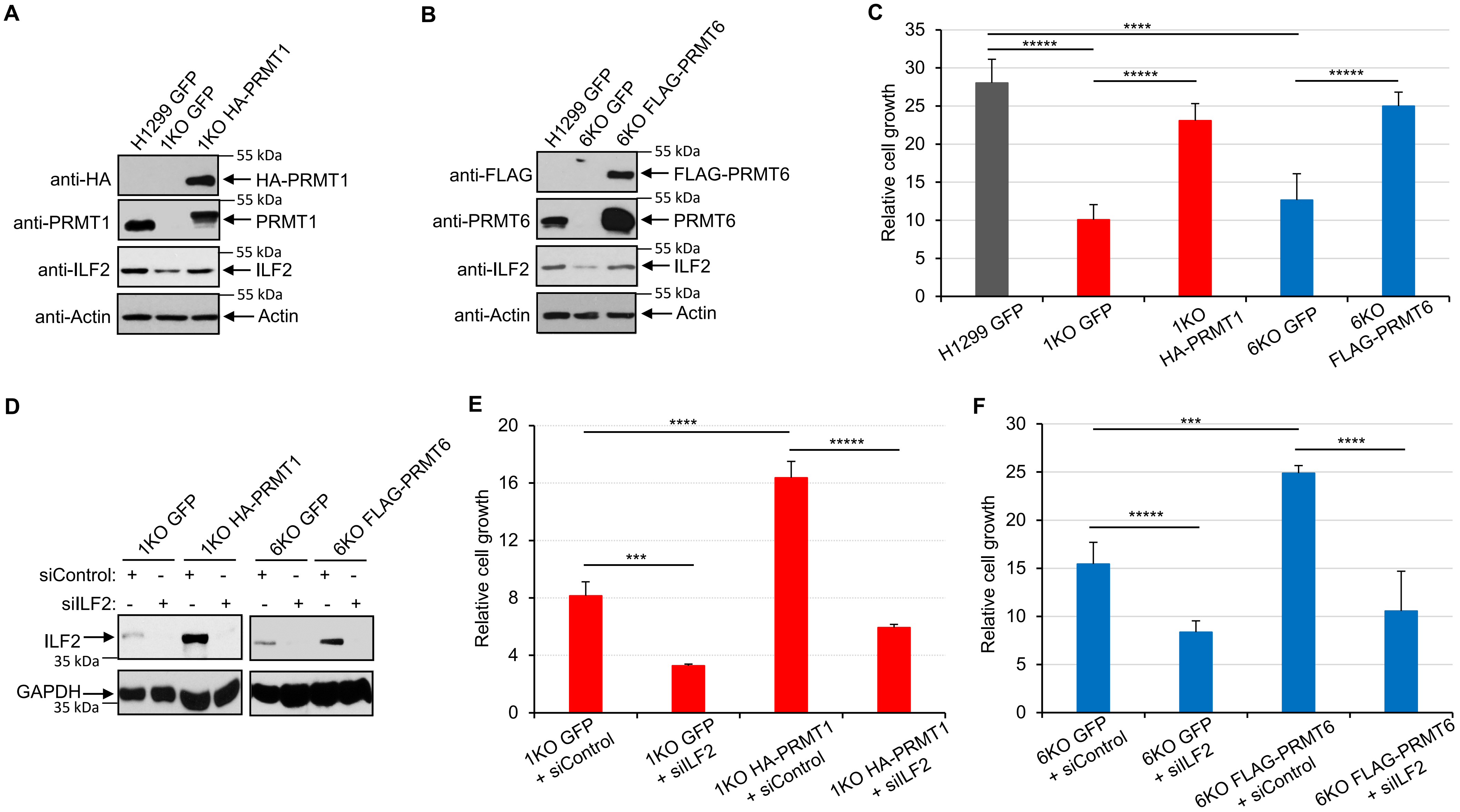

Figure S7. ILF2 promotes cell proliferation, Related to Figure 7. (A) Extracts of H1299 cells transduced with a lentiviral vector expressing GFP or PRMT1 KO cells transduced with lentiviral vectors expressing GFP or HA-PRMT1 were analyzed by immunoblotting using anti-HA, anti-PRMT1, anti-ILF2, or anti-Actin. (B) Extracts of H1299 cells transduced with a lentiviral vector expressing GFP or PRMT6 KO cells transduced with lentiviral vectors expressing GFP or FLAG-PRMT6 were analyzed by immunoblotting using anti-FLAG, anti-PRMT6, anti-ILF2, or anti-Actin. (C) Restoration of cell growth in PRMT1 KO with re-expression of PRMT1 and in PRMT6 KO with re-expression of PRMT6. Data are represented as mean ± SD (one-way ANOVA). (D) ILF2 expression in PRMT1 KO expressing GFP or HA-PRMT1 transfected with siControl or siILF2, or in PRMT6 KO expressing GFP or FLAG-PRMT6 transfected with siControl or siILF2 was analyzed by immunoblotting. (E and F) Growth of transfected cells in (D) was analyzed at day 5. Data are represented as mean ± SD. ***, $P < 0.0005$; ****, $P < 0.00005$; *****, $P < 0.000005$ (one-way ANOVA).
